# Photosynthesis-independent production of reactive oxygen species in the rice bundle sheath during high light is mediated by NADPH oxidase

**DOI:** 10.1073/pnas.2022702118

**Published:** 2021-06-21

**Authors:** Haiyan Xiong, Lei Hua, Ivan Reyna-Llorens, Yi Shi, Kun-Ming Chen, Nicholas Smirnoff, Johannes Kromdijk, Julian M. Hibberd

**Affiliations:** ^a^Department of Plant Sciences, University of Cambridge, Cambridge CB2 3EA, United Kingdom;; ^b^State Key Laboratory of Crop Stress Biology in Arid Areas, Northwest A&F University, Yangling 712100, China;; ^c^College of Life Sciences, Northwest A&F University, Yangling 712100, China;; ^d^College of Life and Environmental Sciences, University of Exeter, Exeter EX4 4QD, United Kingdom

**Keywords:** rice, bundle sheath strands, high light, reactive oxygen species

## Abstract

When exposed to high light, plants produce reactive oxygen species (ROS). In *Arabidopsis thaliana*, local stress such as excess heat or light initiates a systemic ROS wave in phloem and xylem cells dependent on NADPH oxidase/respiratory burst oxidase homolog (RBOH) proteins. In the case of excess light, although the initial local accumulation of ROS preferentially takes place in bundle-sheath strands, little is known about how this response takes place. Using rice and the ROS probes diaminobenzidine and 2′,7′-dichlorodihydrofluorescein diacetate, we found that, after exposure to high light, ROS were produced more rapidly in bundle-sheath strands than mesophyll cells. This response was not affected either by CO_2_ supply or photorespiration. Consistent with these findings, deep sequencing of messenger RNA (mRNA) isolated from mesophyll or bundle-sheath strands indicated balanced accumulation of transcripts encoding all major components of the photosynthetic apparatus. However, transcripts encoding several isoforms of the superoxide/H_2_O_2_-producing enzyme NADPH oxidase were more abundant in bundle-sheath strands than mesophyll cells. ROS production in bundle-sheath strands was decreased in mutant alleles of the bundle-sheath strand preferential isoform of *OsRBOHA* and increased when it was overexpressed. Despite the plethora of pathways able to generate ROS in response to excess light, NADPH oxidase–mediated accumulation of ROS in the rice bundle-sheath strand was detected in etiolated leaves lacking chlorophyll. We conclude that photosynthesis is not necessary for the local ROS response to high light but is in part mediated by NADPH oxidase activity.

## Significance

The ability of plants to initiate systemic responses to local biotic and abiotic stress is well known. One such example takes place after exposure to high-light episodes and involves an initial, local accumulation of reactive oxygen species (ROS) in the region exposed to excessive light. Overexcitation of the photosynthetic apparatus has long been known to lead to ROS production, but whether this is necessary and sufficient is not known. Here, we show that local synthesis of ROS also requires NADPH oxidases located in bundle-sheath strands and is not dependent on photosynthesis. As NADPH oxidases also allow the systemic ROS response, this work identifies a component that unifies both the local and systemic responses of plants to high light.

Under high-light conditions, the capacity for light capture during photosynthesis can exceed use. This can lead to damage, generate signals promoting repair, and also initiate responses allowing acclimation (1–3). One source of damage is an increase in the production of reactive oxygen species (ROS). For example, oxygen photoreduction, largely at Photosystem I, can result in superoxide and hydrogen peroxide. The other source is singlet oxygen, which is formed by the interaction of oxygen with triplet-state chlorophyll in Photosystem II (2, 4, 5). ROS are potentially harmful, with the ability to damage Fe–S proteins, oxidize amino acid residues, and generate further radicals and reactive electrophiles resulting in lipid peroxidation and DNA damage. Therefore, photosynthetic organisms have evolved a variety of mechanisms to minimize overexcitation of the photosystems. These range from transcriptional responses mediated by retrograde signaling between the chloroplast and the nucleus (2, 3, 6–12) to more immediate remodeling of light-harvesting structures to dissipate excess excitation energy (13, 14).

Processes that dissipate energy in excess of that used by the photosynthetic electron-transport chain are collectively known as nonphotochemical quenching (NPQ) mechanisms, and their induction is thought to reduce damage to the photosynthetic apparatus caused by synthesis of ROS (13, 15). As such, the scavenging/antioxidant network to remove ROS and repair damage is complex (1, 16, 17). Notably, although in C_3_ species such as *Arabidopsis thaliana*, mesophyll (M) cells contain the majority of the chlorophyll in a leaf, after exposure to excess light, ROS accumulate preferentially in bundle-sheath cells that surround veins (16, 18–20). In *Arabidopsis*, ROS have been implicated in rapid systemic signaling responses initiated after various abiotic and biotic stresses including high light, heat, wounding, and pathogen attack (21). Such ROS-mediated systemic signaling from a locally perturbed leaf can lead to stomatal aperture being altered in distant leaves, is associated with the hormones abscisic and jasmonic acid, and is dependent on the plasma membrane–localized NADPH oxidase (AtRBOHD and AtRBOHF) in cells of the phloem and xylem (22–26). Grafting experiments indicate that the initial local propagation of ROS is dependent on respiratory burst oxidase homolog (RBOH) proteins as well as increased cell-to-cell transport that is dependent on plasmodesmata-localized proteins 1 and 5 as well as aquaporins and Ca^2+^-permeable channels in the glutamate receptor–like, mechanosensitive small conductance–like, and cyclic nucleotide–gated families (27).

In contrast to C_4_ species in which the role of the bundle sheath in fixing CO_2_ by RuBisCO has been understood for decades (28), this cell type is poorly characterized in C_3_ species. While the bundle sheath of C_3_ plants contains chloroplasts that accumulate starch (29, 30), they are not as numerous as those in the mesophyll, and reducing chlorophyll accumulation in these cells has limited impact on photosynthesis (31). Rather, in *A. thaliana*, the bundle sheath is thought to be specialized in sulfur metabolism, glucosinolate biosynthesis (32–34), and transport of water and solutes in and out of the leaf (32). In particular, stress-responsive activation of aquaporins in bundle-sheath cells are important for the hydraulic conductivity of the whole leaf (35–37). Consistent with this, bundle-sheath cells more generally have been proposed to play a role in maintaining the hydraulic integrity of the xylem (38, 39) and in regulating flux of metabolites in and out of the leaf (40).

To our knowledge, none of these previous studies explain how the bundle sheath of C_3_ plants preferentially accumulates ROS. One possibility is that the supply of atmospheric CO_2_ to cells around the veins is limited, and so inorganic carbon present in the transpiration stream provides CO_2_ to photosynthesis (41, 42). If this were the case, when stomata close, the provision of CO_2_ from the veins could slow activity of the Calvin–Benson–Bassham cycle compared with chlorophyll de-excitation in the light-harvesting complexes (20). Although the proximity of bundle-sheath cells to veins could provide an efficient mechanism to initiate systemic acclimation to high-light stress (16), the mechanism(s) by which ROS accumulate locally in bundle-sheath cells remain unclear, and, to our knowledge, how common this response is beyond *A. thaliana* is not known.

Using rice, we show that the ability of the C_3_ bundle sheath to preferentially accumulate ROS in response to high light is found in the monocotyledons as well as the dicotyledons. We found no evidence that ROS accumulation in the C_3_ bundle sheath was due to limited CO_2_ supply nor to production of H_2_O_2_ from photorespiration—in fact, we detected clear ROS accumulation in bundle-sheath cells of C_4_ species in which photorespiration is essentially abolished. We also found no evidence for an imbalance between transcript abundance of genes encoding components of light-harvesting apparatus and the Calvin–Benson–Bassham cycle. However, we did find that transcripts encoding NADPH oxidases accumulate preferentially in bundle-sheath cells. Pharmacological treatment to block the activity of NADPH oxidases and the mutant alleles for *OsRBOHA* reduced, while overexpression of *OsRBOHA* increased, accumulation of ROS in the bundle sheath of rice. Although accumulation of ROS in the bundle sheath was strongest in green leaves containing light-harvesting apparatus, accumulation was still detected in etiolated leaves.

## Results

### Veins and Bundle-Sheath Cells of Rice Preferentially Accumulate ROS in Response to High Light.

**Fig. 1.**
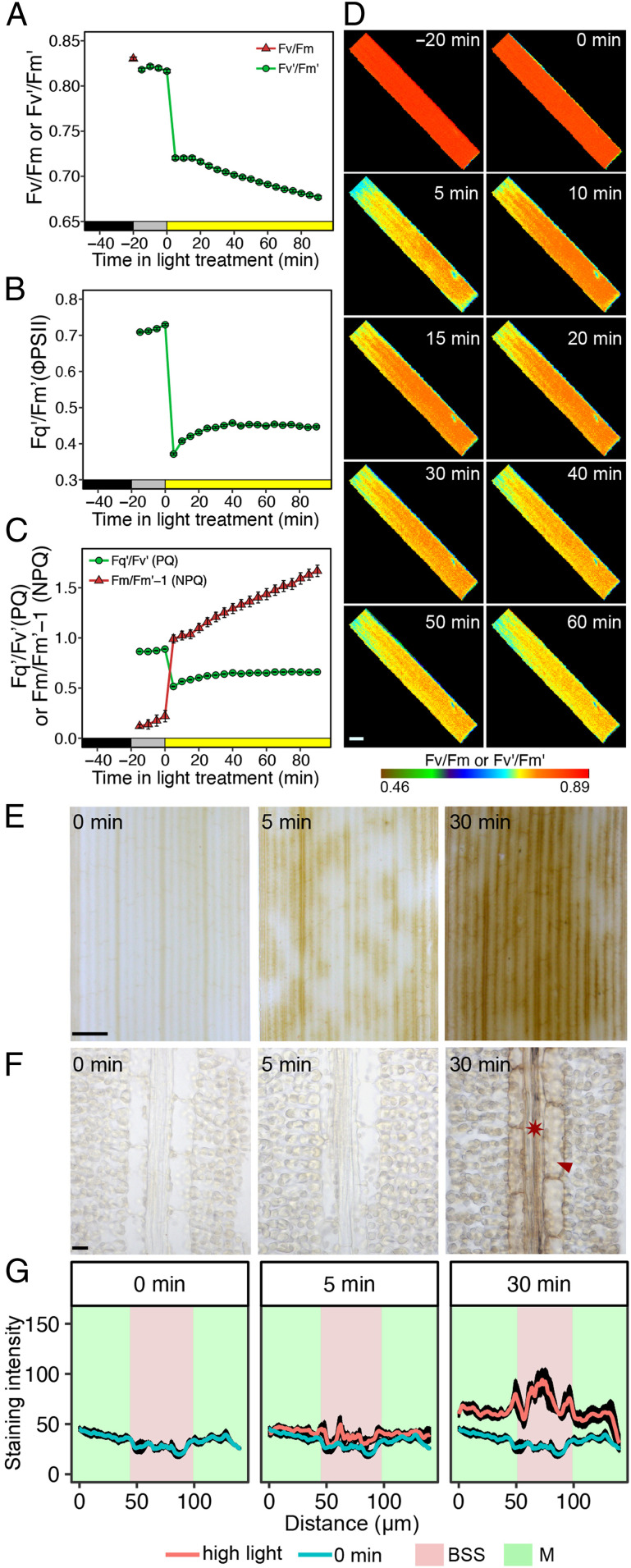
Rice BSS preferentially accumulate the DAB polymerization product in response to high light. (*A*–*C*) Chlorophyll fluorescence parameters associated with dark-adapted leaves being moved into the light intensity of growth for 20 min and then moved to a 10-fold higher intensity of light. (*A*) Dark-adapted F_v_/F_m_ and F_v_’/F_m_’. (*B*) Quantum efficiency of Photosystem II (F_q_’/F_m_’ or ΦPSII) and (*C*) PQ and NPQ. Data shown represent mean and SE from 16 leaves. (*D*) Representative images from the chlorophyll fluorescence imager showing responses were reasonably homogenous across the leaf. (Scale bar: 2 mm.) (*E*) High-light stress led to strong staining from the DAB polymerization product in BSS arranged along the proximal to distal axis of the leaf blade. After 5 min of high light, staining is evident, but at 30 min, it is stronger and more homogenous in these BSS. (Scale bar: 1 mm.) (*F*) Representative image from paradermal sections show that cells accumulating DAB stain are veins (asterisk) and bundle-sheath cells (arrowhead). (Scale bar: 10 μm.) (*G*) Semiquantitation of DAB stain in M cells and BSS. Data are presented as mean (red or blue line) and one SE from the mean, *n* = 4).

Rice leaves were exposed for 90 min to a light intensity 10-fold higher than that used for growth. As expected, this led to a rapid and sustained reduction in the chlorophyll fluorescence parameters F_v_′/F_m_′ and F_q_′/F_m_′ (Fig. 1 *A* and *B*) that report the maximum efficiency of Photosystem II and its operating efficiency, respectively. Over the same period, photochemical quenching (PQ) first decreased and then recovered slowly, while NPQ increased steadily (Fig. 1*C*). Representative images of F_v_′/F_m_′ over this time course indicated that responses of the photosynthetic apparatus to high light were relatively homogenous across the leaf (Fig. 1*D*). Together, these data show that subjecting rice leaves to excess light led to the expected response of Photosystem II efficiency.

We next tested whether preferential accumulation of ROS in bundle-sheath cells as reported in *A. thaliana* (18–20) was detectable in rice. The cytochemical dye 3,3-diaminobenzidine (DAB) is routinely used to detect H_2_O_2_. It reacts with H_2_O_2_ to form a brown polymerization product, the reaction being accelerated by peroxidase (43). In contrast to the relatively homogenous alterations to chlorophyll fluorescence parameters reporting on the activity of Photosystem II (Fig. 1*D*), preferential accumulation of the DAB polymerization product (hereafter referred to as DAB) was detected in patches of longitudinal files of cells after 5 min and then in almost all files of these cells after 30 min of exposure to high-intensity (750 μmol ⋅ m^−2^ ⋅ s^−1^) red light (Fig. 1*E*). Paradermal sections from leaves were generated in order to determine the specific cell types involved, and this showed that, 30 min after exposure to high light, the strongest DAB signal was associated with veins and bundle-sheath cells (Fig. 1*F*). We refer to these cells as bundle-sheath strands (BSS), as they include both the bundle sheath and the vascular strands. Semiquantitation of this signal from multiple sections confirmed that BSS consistently accumulated more DAB than the surrounding M cells (Fig. 1*G*), and more dense sampling indicated that the increase in DAB was first detectable 10 min after the transfer to high light (*SI Appendix*, Fig. S1 *A* and *B*). From around 15 min after the treatment, DAB also increased in M cells, but the signal was always stronger in BSS (*SI Appendix*, Fig. S1 *A* and *B*). We used the ROS-sensitive fluorescent dye 2′,7′-dichlorodihydrofluorescein diacetate (H_2_DCFDA) to provide independent evidence that rice BSS were particularly responsive to high light. In the presence of peroxidases and radicals generated by ROS in living plant cells, H_2_DCFDA produces highly fluorescent 2′,7′-dichlorofluorescein (DCF) (44). Consistent with the results obtained with DAB, when H_2_DCFDA was supplied to rice leaves, high light led to brighter green fluorescence in BSS than in neighboring cells (*SI Appendix*, Fig. S2*A*). To exclude the possibility that H_2_DCFDA had not uniformly penetrated M cells, we supplied a subset of leaves with exogenous H_2_O_2_ (*SI Appendix*, Fig. S2*B*). This led to DCF fluorescence from both mesophyll as well as BSS, indicating that the increased signal in the bundle sheath after exposure to high light was unlikely to be an artifact of incomplete transport into all cells of the leaf or of limitation by peroxidase activity (*SI Appendix*, Fig. S2*B*). Localized staining also rules out direct dye oxidation by light (44). As would be expected, application of dichorophenyl-dimethylurea (DCMU) to block the photosynthetic electron-transport chain at Photosystem II reduced DAB oxidation (*SI Appendix*, Fig. S3 *A* and *B*), while paraquat, which promotes ROS production, led to stronger DAB staining (*SI Appendix*, Fig. S3 *C* and *D*). While the difference in the intensity of DAB staining between BSS and M cells could be partly due to higher peroxidase activity in BSS, we conclude that, as with *A. thaliana*, veins and bundle-sheath cells of rice preferentially accumulate ROS in response to high-light treatment and that this may be a property found in both dicotyledons and monocotyledons.

### Overcapacity in Light Harvesting Compared with the Calvin–Benson–Bassham Cycle Capacity Is Unlikely the Cause of DAB Accumulation in Rice BSS.

**Fig. 2.**
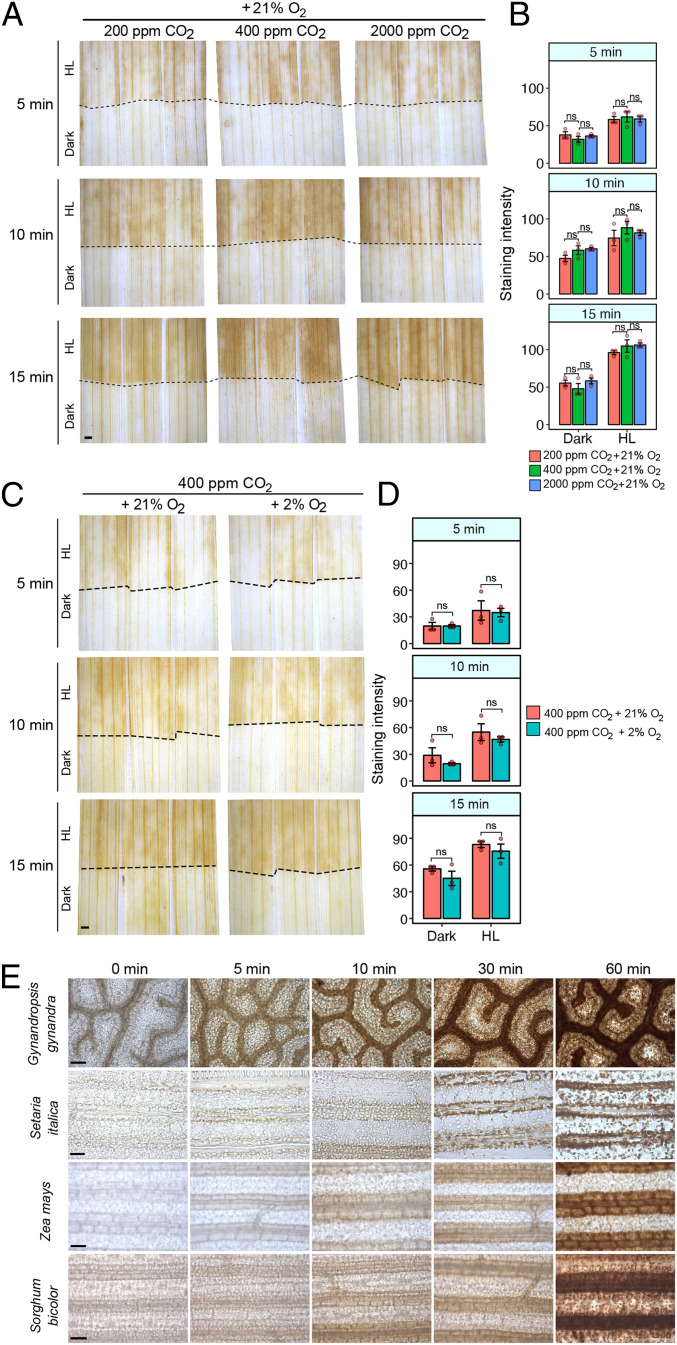
Altering CO_2_ or O_2_ supply has little effect on the high-light response of the rice bundle sheath. (*A*) Neither reducing (200 ppm) nor increasing (2,000 ppm) CO_2_ had a clear effect on DAB staining at 21% O_2_ under high light (750 μmol ⋅ m^−2^ ⋅ sec^−1^ PPFD). The dotted line indicates the boundary between high light (HL) and dark-exposed parts of the leaf. (*B*) Quantitation of DAB staining of leaves exposed to 200, 400, or 2,000 ppm CO_2_. ANOVA indicated no significant statistical difference associated with CO_2_ treatment (*P* = 0.22). (*C*) Inhibition of photorespiration by reducing O_2_ from 21 to 2% at 400 ppm CO_2_ did not abolish H_2_O_2_ accumulation in the bundle sheath under HL (750 μmol ⋅ m^−2^ ⋅ sec^−1^ PPFD). (*D*) Quantification of DAB staining in leaves exposed to 2 or 21% O_2_. ANOVA indicated no significant statistical difference associated with O_2_ treatment (*P* = 0.0933). (*E*) DAB staining leaves of C_4_ *G. gynandra*, *S. italica, Z. mays*, and *S. bicolor* exposed to high light. Although C_4_ species have limited photorespiration in the bundle sheath, DAB staining was still detected in this tissue. (Scale bars: 1 mm in *A* and *C* and 50 μm in *E*.)

Excess excitation energy that cannot be fully utilized by carbon assimilation and other metabolic processes in chloroplasts gives rise to ROS during exposure to high-light intensity (19, 45, 46). The greater production of ROS in the BSS compared with M cells implies a greater capacity for ROS production or a limitation imposed by the CO_2_ assimilation rate. As BSS are distant from stomata and are in contact with fewer intercellular air spaces than M cells, we reasoned that they may be CO_2_ limited. Thus, flux through the Calvin–Benson–Bassham cycle may be constrained compared with the activity of the photosynthetic electron-transport chain. If this were the case, reducing and increasing the CO_2_ concentration around leaves would be expected to respectively enhance and repress preferential DAB staining in BSS. However, we found evidence for neither. High-light exposure at 200 ppm [CO_2_], which would restrict activity of the Calvin–Benson–Bassham cycle, led to preferential but similar DAB staining in the BSS, and 2,000 ppm [CO_2_], which should saturate RuBisCO in the BSS as well as M cells failed to abolish the preferential DAB staining in these cells (Fig. 2 *A* and *B*).

The oxygenation reaction of RuBisCO requires the photorespiratory pathway to detoxify the initial product, phosphoglycolate, and, in so doing, H_2_O_2_ is released by glycolate oxidase in peroxisomes. It is therefore possible that rapid DAB staining in BSS is due to high rates of photorespiration in this cell type. To test this, we reduced the oxygen tension from 21 to 2% but found that this had no clear effect on DAB staining compared with controls (Fig. 2 *C* and *D*). Moreover, when leaves of the C4 species *Gynandropsis gynandra*, *Setaria italica*, *Zea mays*, and *Sorghum bicolor*, which generate up to tenfold higher concentrations of CO_2_ in bundle sheath cells and therefore minimal activities of photorespiration were exposed to high light, DAB still accumulated in these cells over a similar time-course (Fig. 2*E*). It therefore appears unlikely that preferential DAB staining in either C_3_ or C_4_ bundle-sheath cells is caused by H_2_O_2_ produced during photorespiration.

Taken together, our results imply that accumulation of ROS in the bundle sheath of C_3_ and C_4_ plants is unlikely to be caused by the limited capacity of the Calvin–Benson–Bassham cycle compared with the ability to harvest light energy. Furthermore, the data obtained by suppressing photorespiration either transiently in C_3_ leaves or more permanently in C_4_ leaves are inconsistent with the notion that photorespiratory-derived H_2_O_2_ is responsible for the rapid accumulation of ROS in bundle-sheath cells.

### Transcriptome Profiling Indicates a Balanced Expression of Photosynthesis Genes but Elevated Expression of Genes Encoding Enzymes Responsible for Synthesis of ROS in BSS.

**Fig. 3.**
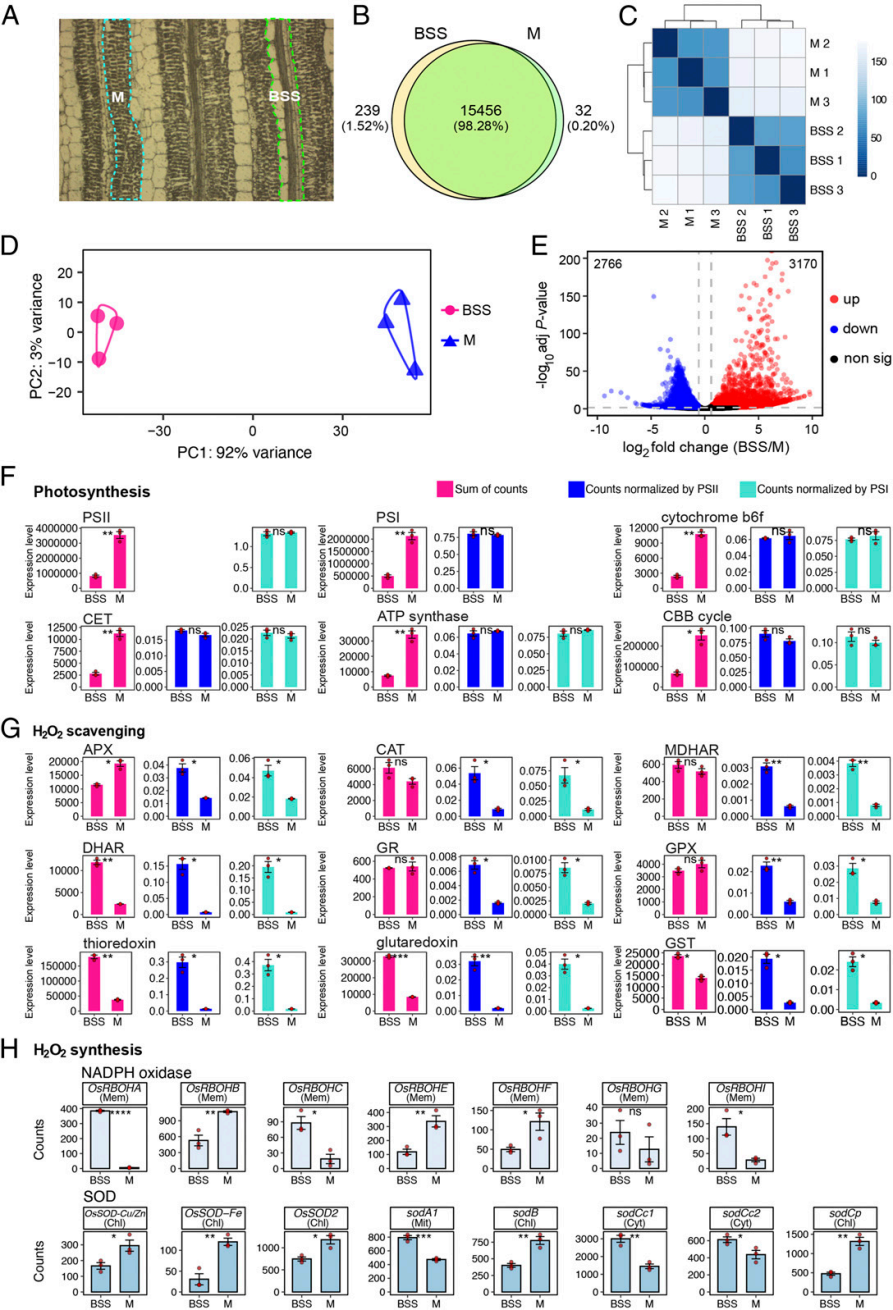
Global analysis mRNA from BSS and M cells indicate a balanced accumulation of transcripts encoding components of the photosynthetic electron-transport chain and the Calvin–Benson–Bassham cycle but that BSS may have greater capacity to synthesize reactive oxygen species. (*A*) BSS and M cells were sampled for RNA using LCM. (*B*) Pie chart summarizing the number of genes expressed in both BSS and M cells. (*C*) Hierarchical dendrogram and (*D*) Principal component analysis indicate that the most variance was associated with tissue type. (*E*) Volcano plot showing the number of differentially expressed genes between BSS and M. (*F*–*H*) Abundance of photosynthesis (*F*), H_2_O_2_ scavenging (*G*), and H_2_O_2_ synthesis (*H*) transcripts. For photosynthesis complexes, the sum of all components of each complex is presented, and, to take into account lower chloroplast content in BSS, these were normalized to either Photosystem I or II. Predicted subcellular localization of each NADPH oxidase (RBOH) and superoxide dismutase (SOD) isoform is annotated (chl = chloroplast, mem = plasma membrane, mit = mitochondrial, cyt = cytoplasm). The *t* tests indicate statistically significant differences (****P < 0.0001, ***P < 0.001, **P < 0.01, *P < 0.05).

To better understand the molecular basis for preferential accumulation of ROS in rice BSS, we carried out RNA-sequencing (RNA-seq) on this tissue. Laser capture microdissection (LCM) was used to obtain messenger RNA (mRNA) from three biological replicates of BSS or M cells derived from leaves that had not received a high-light treatment (Fig. 3*A*). Electropherograms showed that the RNA obtained was of good quality (*SI Appendix*, Fig. S4). In total, over 165 million reads were generated using the Illumina sequencing platform. For each replicate, on average, about 78.5% of reads were mapped uniquely to the Nipponbare reference genome (Dataset S1). To enable comparison between samples, we normalized all read counts with DESeq2, and, to reduce noise, poorly expressed genes with averaged normalized counts of <10 in all samples were removed. This led to a total of 15,727 genes being identified as expressed. Among these, 15,456 genes were expressed in both BSS and M cells, while 239 genes were only expressed in BSS and 32 genes only expressed in M cells (Fig. 3*B*). Hierarchical clustering (Fig. 3*C*) and principal component analysis (PCA; Fig. 3*D*) showed strong clustering between biological replicates from each tissue. Indeed, the PCA showed that 92% of variance between replicates was associated with one major component that mapped onto the tissue from which RNA was isolated (Fig. 3*D*). Using criteria of log_2_ fold change > 0.5 and an adjusted *P* value < 0.05, we defined 3,170 genes as being more highly expressed in BSS and 2,766 as more strongly expressed in M cells (Fig. 3*E* and Dataset S2).

We next analyzed the abundance of transcripts derived from genes known to be associated with the production of ROS. Specifically, we focused on genes encoding the photosynthetic apparatus and enzymes that either scavenge or synthesize ROS. In all cases, to provide an overview of these complex processes, eigengene values were computed to take into account the fact that multiple genes encode these oligomeric protein complexes. Transcripts encoding components of Photosystem II, Photosystem I, the cytochrome *b*_*6*_*f* complex, cyclic electron transport, and the adenosine triphosphate (ATP) synthase were less abundant in BSS compared with M cells (Fig. 3*F* and *SI Appendix*, Fig. S5). However, the mesophyll contains a larger chloroplast compartment (29), so this is to be expected. We thus normalized transcript abundance for each complex to Photosystem II and Photosystem I (*SI Appendix*, Fig. S5). This showed that components of Photosystem II, the cytochrome *b*_*6*_*f* complex, and Photosystem I accumulated stoichiometrically in both cell types (Fig. 3*F*). In other words, there was no clear imbalance in transcripts encoding one part of the photosynthetic electron-transport chain that might lead to impaired function. This was also true for transcripts encoding enzymes of the Calvin–Benson–Bassham cycle (Fig. 3*F*), indicating that the relative capacities of the light-dependent reactions of photosynthesis and the Calvin–Benson–Bassham cycle are balanced similarly in BSS and M cells. These data are consistent with our earlier finding that increasing the CO_2_ concentration around leaves failed to abolish accumulation of DAB staining in the rice bundle sheath (Fig. 2 *A* and *B*).

We also found no evidence from this analysis of transcript abundance that BSS may have a lower ability to detoxify ROS than mesophyll (Fig. 3*G*). After normalization to transcripts for the Photosystems, which would be an expected source of ROS during high-light stress, transcripts encoding enzymes known to scavenge ROS were typically more abundant in BSS compared with M cells (Fig. 3*G*). However, transcripts encoding several NADPH oxidase and superoxide dismutase proteins, which generate superoxide and H_2_O_2_, respectively, were also more abundant in BSS compared with M cells (Fig. 3*H*). In particular, transcripts encoding OsRBOHA, OsRBOHC, and OsRBOHI were considerably more abundant in BSS compared with M cells. qPCR confirmed these findings (*SI Appendix*, Fig. S6). These data imply that the rapid increase in ROS in BSS after exposure to high light is unlikely associated with a limited ability to dissipate energy associated with the photosynthetic electron chain, but it could be due to higher basal activities of proteins that synthesize ROS.

### NADPH Oxidase Activity Mediates the High-Light Response in Both Green and Etiolated Leaves.

**Fig. 4.**
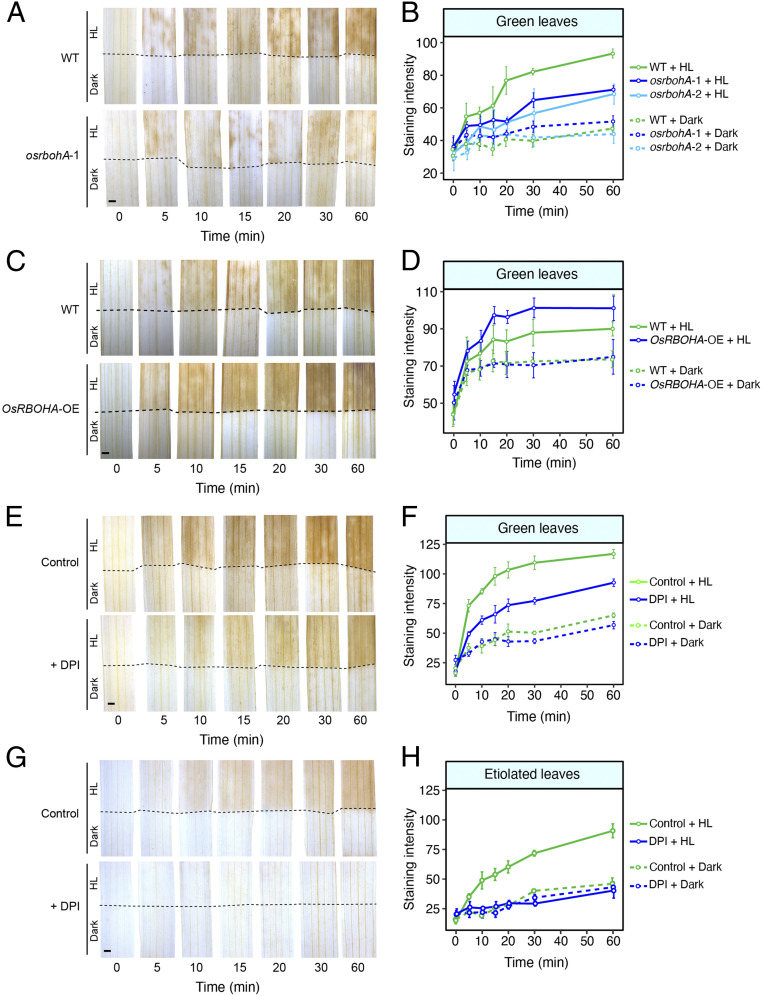
Inhibiting and increasing NADPH oxidase reduces and increases DAB staining, respectively, in BSS of rice leaves. (*A*) Representative images showing that the mutant allele 1 of *OsRBOHA* (*osrbohA*-1) has reduced DAB staining compared with the wild type under high-light treatment. (*B*) Semiquantitation of DAB staining over time in green leaves from either the wild type or *osrbohA*-1 and *osrbohA*-2. (*C*) Overexpression of *OsRBOHA* increased DAB staining in BSS after high-light treatment. (*D*) Semiquantitation of DAB staining over time in green leaves from either the wild-type or *OsRBOHA*-overexpression line. (*E*) DPI, a plasma membrane NAD(P)H oxidase inhibitor, partially inhibits DAB staining in BSS of green leaves. (*F*) Semiquantitation of DAB staining over time in the control compared with DPI and high light–treated green leaves. (*G*) DPI completely inhibits DAB staining in etiolated leaves. (*H*) Semiquantitation of DAB staining over time in the control and DPI high light–treated etiolated leaves. In all cases, the lower portion of the leaf (below the dotted line) was covered to provide an in-leaf control over the time course. ANOVA showed DAB staining was reduced after DPI treatment in green leaves (*P* < 0.001), etiolated leaves (*P* < 0.001), *OsRBOHA* mutant allele *osrbohA*-1 (*P* < 0.01), and *osrbohA*-2 (*P* < 0.0001), and increased in *OsRBOHA* overexpressors (*P* < 0.05). (Scale bars: 1 mm.)

To test whether NADPH oxidase activity is important for ROS accumulation in BSS of rice, we used two mutant alleles, a previously reported overexpression line for the *OsRBOHA* gene that encodes the major RBOH isoform NADPH oxidase A in rice (47), and inhibitors to block its activity. It should be noted that individual mutant alleles are unlikely to completely abolish the ROS response, as there are multiple isoforms of NADPH oxidase, and also that the inhibitors are not completely specific to NADPH oxidase and can therefore impact other oxidases. Green leaves of the mutant alleles *osrbohA-1* and *osrbohA-2* showed reduced DAB staining (Fig. 4 *A* and *B* and *SI Appendix*, Fig. S7 *A*–*F*), and the overexpressor of *OsRBOHA* showed increased DAB staining in rice BSS compared with controls (Fig. 4 *C* and *D* and *SI Appendix*, Fig. S7 *G* and *H*). Two commonly employed inhibitors of flavin-linked enzymes, diphenyleneiodonium chloride (DPI) (48) and imidazole (49), reduced ROS production in BSS after the imposition of light stress (Fig. 4 *E* and *F* and *SI Appendix*, Fig. S8 *A* and *B*). When leaves were treated with the calcium channel inhibitor LaCl_3_ or the Ca^2+^ chelator EGTA, DAB staining was inhibited after high-light treatment (*SI Appendix*, Fig. S9 *A*–*D*). These data indicate that activation of OsRBOHA is dependent on calcium, and they are consistent with the ROS wave being linked to calcium signaling (50).

We next exposed etiolated leaves of *rbohA* mutant alleles to high light and found that, despite the fact that they lacked chlorophyll, DAB accumulation was reduced compared with controls (*SI Appendix*, Fig. S10 *A* and *B*). It is possible that the remaining DAB staining is associated with other RBOH proteins that remain active, and, consistent with this hypothesis, when etiolated leaves from wild type were exposed to the RBOH inhibitors DPI and imidazole, DAB accumulation was no different from the tissue kept in the dark (Fig. 4 *G* and *H* and *SI Appendix*, Fig. S11 *A* and *B*). In summary, the incomplete abolition of DAB accumulation in green leaves subjected to high light and treated with DPI and imidazole (Fig. 4 *E* and *F* and *SI Appendix*, Fig. S8 *A* and *B*) is consistent with some ROS being generated from photosynthesis. However, as DAB was oxidized in response to high light in etiolated leaves, this also means a second local source of ROS exists, and, as this is reduced in *rbohA* mutants and removed completely by DPI and imidazole, this must be generated by RBOH proteins. Overall, our data are therefore consistent with ROS being produced by photosynthetic but also nonphotosynthetic pathways. qPCR on RNA isolated from etiolated leaves confirmed that, in etiolated leaves, transcripts derived from *OsRBOHA*, *OsRBOHC*, and *OsRBOHI* genes as well as superoxide dismutase genes were more abundant in BSS than in M cells (*SI Appendix*, Fig. S12). These findings contrast with analysis of a mutant allele (*osrbohB*) and overexpressor of *OsRBOHB*, which was preferentially expressed in M cells (Fig. 3*I*). In these lines, no impact on DAB staining during high-light treatment was detected (*SI Appendix*, Fig. S13). We thus conclude that bundle sheath–preferential *RBOH* genes, and, in particular, *OsRBOHA*, are important in mediating the response to high light in BSS of rice.

## Discussion

In the C_3_ species *A. thaliana*, bundle-sheath cells accumulate ROS in response to excess light (16, 18–20). Recently, the systemic response associated with this local accumulation of ROS has been shown to be dependent on NADPH oxidases located in the phloem and xylem (22–26). Here, we focused on local processes associated with initiation of this system response. In so doing, our data show that a similar local response is detected in BSS of rice, a C_3_ species from the monocotyledons. Moreover, our analysis indicates that this phenomenon is not associated with the C_3_ pathway, but that it also takes place in BSS from leaves of C_4_ plants. This was the case in C_4_ *G. gynandra*, which is sister to the Brassicaceae, and also in the C_4_ grasses maize, sorghum, and *Setaria*. These findings imply that the bundle sheath from dicotyledons and monocotyledons of both C_3_ and C_4_ species fulfills a role in sensing and responding to excess light.

High light–induced ROS was not abolished by decreasing photorespiration in rice and could be seen in the BSS of C_4_ species that lack photorespiration. Therefore, photorespiratory H_2_O_2_ production in peroxisomes via glycolate oxidase is not involved. The possibility that an imbalance between light harvesting and photosynthetic electron transport and use of excitation energy by the Calvin–Benson–Bassham cycle in bundle-sheath cells was not supported by the relative expression of genes encoding photosynthetic components. After normalization, these have similar relative expression in both cell types. This finding is consistent with analysis of the bundle sheath in C_3_ *A. thaliana*, in which the photosynthetic apparatus is assembled and functional (29, 31) despite lower levels of photosynthesis gene expression (32). We also failed to collect any convincing physiological evidence that ROS production in the rice bundle sheath was associated with restricted activity of the Calvin–Benson–Bassham cycle, as might be expected if provision of CO_2_ to this tissue distant from the stomata was limiting. The accumulation of ROS was neither accentuated nor ameliorated when intercellular CO_2_ concentrations were reduced or increased, respectively.

In contrast to a purely photosynthetic origin of ROS in BSS, we present evidence that plasma membrane–localized NADPH oxidase plays a significant role. NADPH oxidase catalyzes extracellular reduction of oxygen, forming superoxide in the apoplast. Membrane impermeable–superoxide dismutates rapidly, producing H_2_O_2_, which is proposed to enter the cytosol through aquaporins. NADPH oxidase has numerous isoforms and is activated rapidly following numerous stimuli (51). Apoplastic ROS production in *A. thaliana* bundle-sheath cells in response to high light has been reported (20), although NADPH dependence was not established. We found that three NADPH oxidase isoforms (*OsRBOHA*, *OsRBOHC*, and *OsRBOHI*) were more highly expressed in rice BSS compared with M cells. We were not able to confirm that NADPH oxidase activity is strongest in BSS, so it is possible that they produce the ROS in the mesophyll and that aquaporins then transport ROS to the bundle sheath. If true, this would be analogous to the role of aquaporins in mediating the long-distance high-light systemic signal (27). However, it does appear that specific isoforms of NADPH oxidase are responsible for this high-light response. For example, an overexpressor of *OsRBOHB*, which is well known in rice’s defense against *Magnaportha oryzae* (52–54), and whose transcripts were more abundant in M cells, showed no effect on ROS accumulation under high light. In contrast, mutant alleles of *OsRBOHA* had reduced DAB staining, and an *RBOHA* overexpressor showed increased production of ROS in the bundle sheath. Furthermore, the observation that etiolated leaves have high light–induced ROS accumulation provides key evidence that it can be independent of photosynthesis and that it supports a role for NADPH oxidase.

This local response to high-light stress and accumulation of ROS takes place in etiolated leaves that lack photosynthetic apparatus. These data therefore suggest that the systemic response (25) does not require photosynthesis to be initiated. How NADPH oxidase is activated by light in etiolated leaves will need to be investigated further in the future. One possibility could be photoreceptor-mediated NADPH oxidase activation. Cryptochrome generates superoxide upon exposure to blue light, and this has been proposed to contribute to part of its signaling role (55). However, in *A. thaliana*, the ROS wave allowing systemic signaling after high-light stress is dependent on red light (56). Moreover, while *phyB* and *phyAphyB* mutants lacked both local and systemic stomatal responses to high light, *phyA* showed a local response but lacked the systemic response (56). It therefore appears that PhyA may be required for the systemic stomatal response. As we used red light in our experiments on rice, this indicates that both the local accumulation of ROS and the subsequent ROS wave necessary for any systemic response may be unified by a red light– and NADPH oxidase–dependent pathway in BSS. As the local accumulation of ROS was not completely abolished in *rbohA* mutants and it was responsive to both DCMU and paraquat, the results also suggest that part of the light-induced ROS derive from photosynthesis. Given that previous work has shown that chloroplasts release H_2_O_2_ in the light and that this could provide a high-light signaling mechanism (51, 57), the possible differences in the function of chloroplasts and NADPH oxidase–derived ROS require investigation.

NADPH oxidase has previously been implicated in ROS responses induced by biotic and abiotic stresses. In response to a localized stress event, an NADPH oxidase–dependent ROS wave propagates between cells to initiate systemic responses in tissues distant from the original stress (17, 24, 58, 59). While we did not aim to investigate components underpinning such a wave or downstream responses, our findings have some relevance to the role of NADPH oxidase in ROS production. The data imply that rice *OsRBOHA* plays a role in the local production of ROS in BSS. RBOH proteins are encoded by 10 and 9 genes in *A. thaliana* and rice, respectively (60), and, as the phylogenetic distance between *A. thaliana* and rice means that direct orthologs are not always found, the function of these proteins may have diverged. However, *OsRBOHA* from rice, which appears to play an important role in ROS accumulation in the bundle sheath in response to high light, is in the same orthogroup as *AtRBOHF* from *A. thaliana* (60). Interestingly, compared with the whole leaf, transcripts of *AtRBOHF* were more abundant in bundle-sheath cells (*SI Appendix*, Fig. S14*A*; ref. 32). Moreover, *AtRBOHF* was recently reported to be required for systemic ROS signaling at the vascular bundles of *Arabidopsis* under high light (25). Thus, it is possible that these orthologs fulfill similar functions in the BSS of both species. However, several lines of evidence indicate that multiple pathways may synthesize and propagate ROS production in response to stresses. For example, in *A. thaliana*, production of ROS takes place in BSS in leaves subjected to high-light stress (16, 18–20), while, in distant leaves, ROS production is preferentially detected in M cells (21, 59). Moreover, differential changes in the stomatal aperture of leaves subjected to stress compared with distant nonstressed leaves support a model in which two different signals are involved in systemic stomatal responses. One is abscisic acid based and associated with the vascular system (61–64), and another uses ROS and travels through the plant (22). In fact, after a dark-to-light transition in *A. thaliana*, local changes to stomatal aperture were not dependent on *AtRBOHD*, but those changes associated with the systemic response were. *AtRBOHD* from *A. thaliana* is orthologous to *OsRBOHI* from rice (60), and, in both species, transcripts appear to be more abundant in BSS (*SI Appendix*, Fig. S14*B*). This finding implies that the role of these two proteins may have been conserved, since they diverged from the last common ancestor of rice and *A. thaliana*. Downstream effects of ROS accumulation in the bundle sheath will require additional experimentation. One important advance in this area has been the ability to noninvasively monitor ROS either with reporters such as HyPer2 and roGFP-Orp1 (57, 65) or with the addition of fluorescent dyes to intact leaves to allow time-course analysis in locally stressed as well as distantly responding tissues (59). Being able to apply these noninvasive approaches to tissues, such as the bundle sheath, that are deep in the leaf and therefore challenging to image will be informative in the future.

## Materials and Methods

### Plant Materials and High-Light Stress.

Rice (*Oryza sativa* L.) plants were grown for 2 wk in compost in a growth chamber with a 16-h photoperiod with a photosynthetic photon flux density (PPFD) of 75 μmol ⋅ m^−2^ ⋅ s^−1^, day–night temperatures of 28 and 26 °C, respectively, and relative humility of 60%. In all experiments, unless otherwise stated, IR64 was used. Mutant lines (*osrbohA-1*, *osrbohA-2*, and *osrbohB*) and overexpression lines for *OsRBOHA* and *OsRBOHB* as well as their background (Nipponbare) were grown under the same conditions. Etiolated plants were obtained by growing the plants in compost in the dark at 28/26 °C for 2 wk.

In all cases, the middle part (∼2 cm) of the second leaf was taken and infiltrated with dye solution (see *Detection of ROS*). Leaves were then subjected to 750 μmol ⋅ m^−2^ ⋅ s^−1^, which represented a 10-fold increase above that of growth. High light was provided using a Clark-type oxygen electrode (LD2/3 oxygen electrode chamber) connected to an Oxylab control unit (Hansatech Instruments Ltd.) and temperature was maintained at 28 °C using a water tank. The top half of the leaves were illuminated by a liquid electronic display red-light source (Hansatech LH36-2), while the bottom half of the leaf was covered with tin foil to keep it in the dark.

### Chlorophyll Fluorescence Measurements.

Chlorophyll fluorescence measurements were performed using a chlorophyll fluorescent imaging system (CF Imager, Technologica Ltd.). The ∼2-cm leaf strips of the middle part of the second leaves were detached and floated on water in a 25-well square dish and transferred immediately into the CF Imager. The application of preprogrammed regimes of actinic growth light exposure times, dark periods, saturating light pulses, and the calculation and imaging of the parameters F_v_/F_m_, F_q_’/F_m_’, F_v_’/F_m_’, and NPQ (66) were performed using the manufacturers software FluorImager.

### Detection of ROS.

H_2_O_2_ production in the cells was detected by staining with DAB (18, 67). The middle 2 cm from the second leaves were cut and soaked in 5 mM DAB solution (pH 5.0) with 0.01% Tween 20. After shaking in a dark incubator at 28 °C for 2 h, leaves were briefly dried with tissue paper, placed in a Hansatech LD2/ 3 electrode leaf chamber, and subjected to 750 μmol ⋅ m^−2^ ⋅ s^−1^. Sampling was undertaken at 0, 5, 10, 15, 20, 30, 40, 50, and 60 mins. Prior to imaging, chlorophyll was removed from the leaves by soaking in ethanol:acetic acid (3:1) in a 70 °C water bath for 1 h and then immersed in 70% (vol/vol) ethanol for 24 h.

H_2_O_2_ production was also detected with H_2_DCFDA (Sigma-Aldrich). H_2_DCFDA was dissolved in dimethyl sulfoxide (DMSO) to 20 mM and then diluted with H_2_O to a final concentration of 10 μM for infiltration. Infiltration was performed as previously described (18, 67). In short, H_2_DCFDA was fed through the transpiration stream under low light (20 μmol ⋅ m^−2^ ⋅ s^−1^) for up to 12 h to ensure absorption throughout the leaf. After infiltration, leaves were then clamped in the Hansatech LD2/3 electrode chamber and exposed to PPFD of 750 μmol ⋅ m^−2^ ⋅ s^−1^ for 0, 30, and 60 min. Prior to confocal laser scanning microscopy, the middle vein of the leaf was imaged after generating a paradermal section by hand. To confirm whether H_2_DCFDA penetrated into the whole leaf, 30% (wt/vol) H_2_O_2_ solution was diluted to 100 mM with 10 μM H_2_DCFDA and used to infiltrate leaves under the same conditions under the conditions described in *Plant Materials and High Light Stress*. Leaves infiltrated with ddH_2_O at the same condition were used as controls. After infiltration, green fluorescence was assessed by confocal laser scanning microscopy.

To test the effects of different CO_2_ and O_2_ contents on H_2_O_2_ products, DAB-infiltrated leaves were subjected to the high-light treatment as described in *Plant Materials and High Light Stress*, using air mixtures with different concentrations of CO_2_ controlled by an open gas exchange analyzer (LI-6400, LI-COR Biosciences) connected in line to the Hansatech LD2/ 3 electrode leaf chamber. For measurements at 2% O_2_ concentration, the LI-6400 air inlet was connected to a gas cylinder with premixed 2% O_2_ in N_2_.

Pharmacological treatments with DCMU, paraquat, diphenyliodonium, imidazole, LaCl_3_, or EGTA were added to the DAB solution and infiltrated into leaves for 2 h before high-light treatment. DCMU was dissolved in ethanol to 20 mM and then diluted to 100 μM with a 5 mM DAB solution (pH 5.0, with 0.01% [vol/vol] Tween 20). Paraquat was dissolved in water to 20 mM and then diluted to 100 μM with a 5 mM DAB solution (pH 5.0, with 0.01% [vol/vol] Tween 20). DPI chloride was dissolved in DMSO to 100 mM and then diluted to 100 μM with a 5 mM DAB solution (pH 5.0, with 0.01% Tween 20). Imidazole was dissolved in DMSO to 1 M and then diluted with 5 mM DAB solution (pH 5.0, with 0.01% Tween 20) to 20 mM. LaCl_3_ was dissolved in deionized water to 0.5 M and diluted with 6.25 mM DAB solution (pH 5.0, with 0.01% [vol/vol] Tween 20) to 100 mM (with final concentration of DAB to 5 mM). EGTA was dissolved in 1 M KOH to 0.5 M (adjusted pH to 5.0) and then diluted with 6.25 mM DAB (pH 5.0, with 0.01% [vol/vol] Tween 20) to 100 mM. All controls were subjected to 5 mM DAB (pH 5.0, 0.01% [vol/vol] Tween 20).

### Paradermal Sectioning of Paraffin-Embedded Tissue.

Leaves used for paradermal sectioning had been soaked with DAB and treated with high light, and then chlorophyll was removed. Samples were cut into small pieces of ∼5 mm and dehydrated in an ethanol series consisting of 70% (vol/vol) for 30 min, 85% (vol/vol) for 30 min, 95% (vol/vol) for 30 min, 100% (vol/vol) ethanol twice for 30 min, then twice in 100% (vol/vol) Histo-Clear II for 60 min each, followed by two times in paraffin wax for 60 min at 60 °C. Melted paraffin wax was poured into 9-cm Petri dishes and cut into small blocks once the wax was cool. For paradermal sectioning, blocks were trimmed so that the surface of the leaf was parallel to the surface of the block. The 10-µm sections were obtained using a rotary microtome, floated in a 60 °C water bath, and then mounted onto clean glass slides to dry overnight in an incubator set at 42 °C. Dehydration of the sections was performed as follows: 100% (vol/vol) Histo-Clear II twice for 10 min each, 100% ethanol twice for 5 min each, 95% ethanol (vol/vol), 70% (vol/vol) ethanol, 50% (vol/vol) ethanol, and 30% (vol/vol) ethanol for 2 min each. Slides were then rinsed with deionized H_2_O, drained, and then 30% (vol/vol) glycerol was added prior to a coverslip.

### LCM.

For LCM, leaf samples were cut into ∼5-mm pieces and fixed in 100% (vol/vol) ice-cold acetone at 4 °C overnight. The next day, samples were dehydrated and embedded with Steedman’s polyester wax (68). Blocks were sectioned using a rotary microtome to 8-μm thickness and then floated on Arcturus polyethylene naphthalate (PEN) membrane slides (Thermo Fisher Scientific) with diethylpyrocarbonate (DEPC)-treated water. The water was dried using tissue paper, and slides were stored at −20 °C and used within 12 h after sectioning. Prior to LCM, slides were washed in 100% (vol/vol) ethanol for 5 min and then air-dried for 5 min. LCM was performed using the ArcturusXT Laser Capture Microdissection System (Thermo Fisher) according to the manufacturer’s instructions. Mesophyll cells and bundle sheath stands were collected on the CapSure Macro LCM Caps (Thermo Fisher Scientific) and immediately treated with extraction buffer (from Arcturus Picopure RNA extraction kit, Thermo Fisher Scientific) at 42 °C for 30 min and then stored at −80 °C.

### RNA Extraction and qPCR.

Total RNA from LCM-harvested M and bundle-sheath cells was extracted using the Arcturus Picopure RNA extraction kit with on-column DNaseI treatment according to the manufacturer’s protocol. complementary DNA (cDNA) synthesis for LCM samples was performed using the TruSeq RNA Library Preparation Kit version 2 (RS-122-2001, Illumina). Total RNA from whole leaves was extracted from 100 to 200 mg fully expanded leaves using Triazol reagent (Sigma-Aldrich) according to the manufacturer’s instructions. Superscript II reverse transcriptase (18064-022, Thermo Fisher Scientific) was used for cDNA synthesis of whole leaves. qPCR was performed with the Bio-Rad CFX384 Real-Time PCR system using the SYBR Green Jumpstart Taq ReadyMix (S4438-100RXN, Sigma-Aldrich) with the following PCR conditions: 94 °C for 5 min, 40 cycles of 94 °C for 10 s, and 60 °C for 1 min. Relative gene-expression levels were calculated using the 2^-ΔΔCT^ method (69) and normalized with *OsUBQ5.* Gene-specific primer sequences are listed in Dataset S3. For each gene, three biological and three technical replicates were performed.

### RNA-Sequencing Library Preparation and Data Processing.

RNA integrity and the quality of LCM samples were assessed using the 2100-Bioanalyzer (Agilent Technologies) with an Agilent Bioanalyser RNA 6000 Pico assay and QuBit (Thermo Fisher Scientific), respectively. Only samples with RNA integrity numbers ≥4.4 were selected for the final sample cohort. A total of ∼50 to ∼150 ng starting RNA from ∼12 to ∼15 paradermal sections of each replicate were used for RNA-seq library construction using the QuantSeq 3′ mRNA-Seq Library Prep Kit (Lexogen) according to the manufacturer’s recommendations. cDNA libraries were assessed using the 2100-Bioanalyzer before 100-bp single-end sequencing using the NextSeq500 (Illumina) system at the Department of Biochemistry Sequencing Services at the University of Cambridge based on standard protocols. Three biological replicates were conducted for each cell type. Data processing was performed using custom scripts. Briefly, raw reads were processed using Trimmomatic (70), mapped to the reference rice transcriptome genome (MSU7.0, rice.plantbiology.msu.edu/index.shtml), and quantified using Salmon (71). Differential-expression analysis was performed using DESeq2 (72). Stringent criteria with log_2_ fold change (log_2_FC) > 0.5 and adjusted *P* value (padj) < 0.05 were used to screen the differentially expressed genes between BSS and M cells. Plots were generated with custom scripts in RStudio using the package ggplot2. Three biological replicates for each cell type were performed.

### Identification of the Mutant Lines and Generation of *OsRBOHB*-Overexpression Plants.

The transfer DNA (T-DNA) insertion mutants *osrbohA*-1 (Tos17 ID: NF1015), *osrbohA*-2 (Tos17 ID: ND3303), and *osrbohB* (Tos17 ID: NF5029) were obtained from the Rice Tos17 Insertion Mutant Database (https://tos.nias.affrc.go.jp/). Homozygous plants for T-DNA insertions were identified by PCR-based genotyping. Primer sequences used for genotyping and RT-PCR are listed in Dataset S3. To generate the *OsRBOHB*-OE plants, the full-length coding region of *OsRBOHB* was amplified from the first-strand cDNA of Nipponbare and inserted into vector pCAMBIA1301 under the control of the ubiquitin promoter. The resultant construct was transformed into Nipponbare by *Agrobacterium*-mediated transformation. The primers used for the construct are listed in Dataset S3.

### Imaging.

Intact leaves were imaged with Leica m165 FC microscopy. The paradermal sections were imaged with Olympus BX41 microscopy. In common with all other assays based on insoluble products, DAB staining does not follow the Beer–Lambert Law. Thus, to provide insight into the extent of the staining, semiquantification of DAB-staining intensity was undertaken with ImageJ (Fiji build, version 1.52q, NIH). To measure color intensity across paradermal sections, each image was imported into ImageJ and the target area selected using the rectangle tool. This was followed by selecting “edit” and then “invert” to create a reversed image. Using the “analyze” and “plot profile” tools, a column average was obtained, where the *x*-axis represented horizontal distance through the selection and the *y*-axis the vertically averaged pixel intensity. Finally, the data were exported. To measure intact leaves, the same method was used. Confocal micrographs for detecting the DCF fluorescence were taken using a Leica SP8 confocal microscopy (excitation 488 nm, barrier 515 to 555 nm).

### Statistical Analysis.

All statistical analyses were conducted in R (version 3.6.3). A two-way ANOVA was used to assess statistical differences in DAB staining between BSS and M cells after a time course of high-light treatment as well as the effects of different concentrations of CO_2_ and O_2_ concentrations on H_2_O_2_ production under high-light treatment. A three-way ANOVA was used for statistical analysis on the effects of different concentrations of CO_2_ and O_2_ on H_2_O_2_ production under high-light treatment and was also used to assess the data in experiments in which the inhibitors DCMU, paraquat, DPI, imidazole, LaCl_3_, and EGTA were used, and it was also used to compare H_2_O_2_ production of *osrbohA*-1, *osrbohA*-2, *OsRBOHA*-OE, and its wild type over a time course of high-light treatment. Tukey’s honest significant difference test was performed for multiple pairwise comparisons between the means of groups. Levene’s test was used to check the homogeneity of variances, and the Shapiro–Wilk test was used to check the normality assumption. Unpaired *t* tests were performed to compare differences in transcript abundance between BSS and M cells and were also used to compare the differences in DAB staining between wild-type and *OsRBOHB*-overexpression lines and mutants under dark/high light conditions.

## Supplementary Material

Supplementary File

Supplementary File

Supplementary File

Supplementary File

Supplementary File

## Data Availability

Code associated with this manuscript and the underlying data required to generate plots are available in the Github repository: https://github.com/hibberd-lab/Xiong_High-light-response-of-the-rice-bundle-sheath. Raw sequencing data files are deposited in The National Center for Biotechnology Information (PRJNA673407). Accession numbers are shown in Dataset S4.
