# Supplementary material for: Photosynthesis-independent production of reactive oxygen species in the rice bundle sheath during high light is mediated by NADPH oxidase
Source: Proc Natl Acad Sci U S A. 2021 Jun 21;118(25):e2022702118. doi: 10.1073/pnas.2022702118 (PMC8237631; doi:10.1073/pnas.2022702118)
Supplement: Supplementary File [file pnas.2022702118.sapp.pdf]

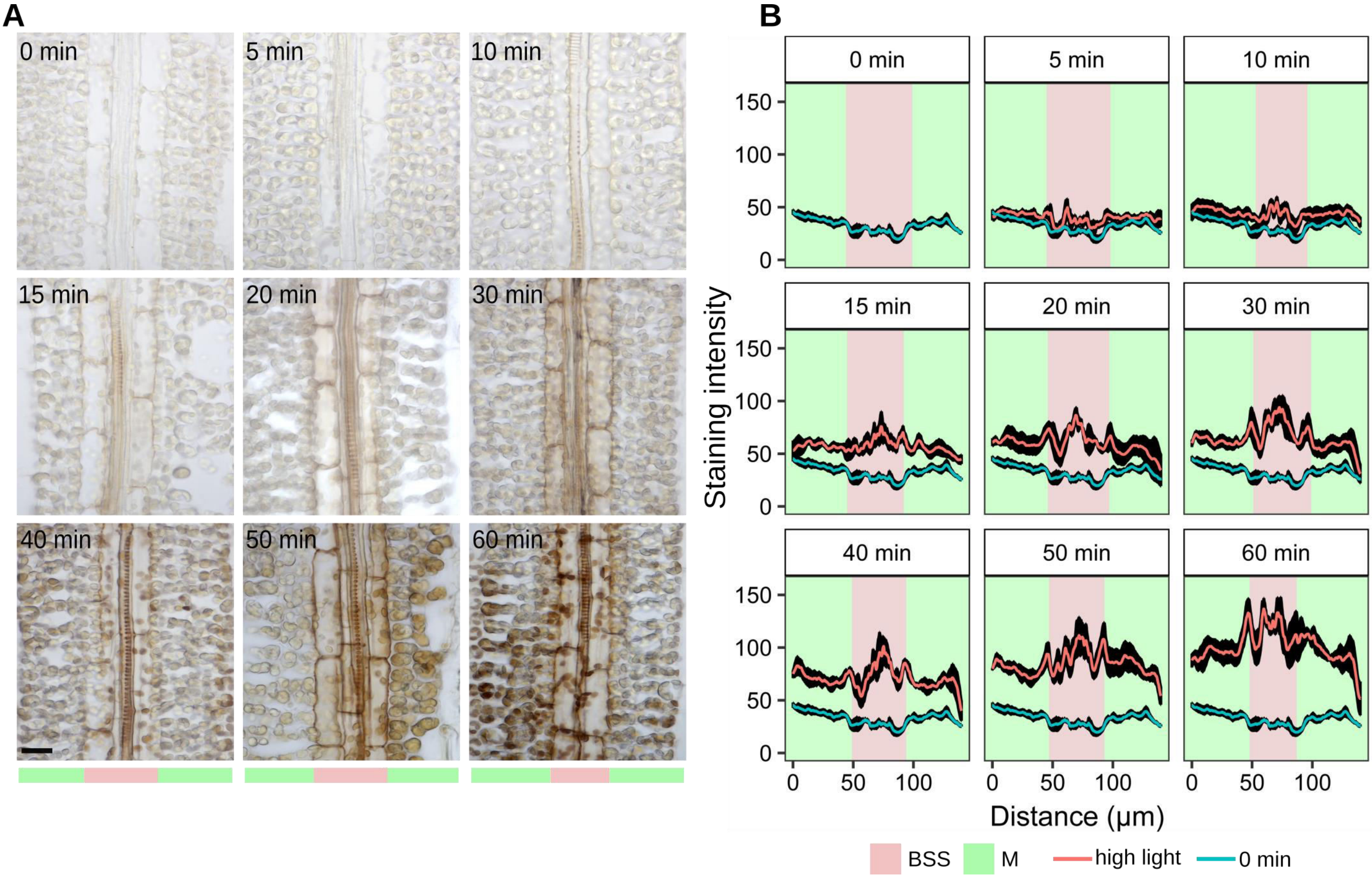

Figure S1. DAB staining of paradermal sections from rice leaves exposed to high light. (A) Representative images of leaves exposed to 750 µmol m^{-2} s^{-1} photon flux density for 0, 5, 10, 15, 20, 30, 40, 50 or 60 minutes. Brown stain indicates the formation of the polymerization product formed in the presence of reactive oxygen species. Scale bar denotes 10 µm. (B) Semi-quantitation (arbitrary units) of DAB staining from paradermal sections illustrating the extent to which reactive oxygen species are first detected in vein and bundle sheath strands (BSS) versus mesophyll (M) cells. The x-axis represents a scan from left to right across the paradermal sections. The y-axis depicts grey values extracted from paradermal sections. Data are presented as mean (red or blue line) and one standard error from the mean (n = 4). The response of bundle sheath strands to high light was greater than that of mesophyll cells (Two-way ANOVA $p<0.001$).

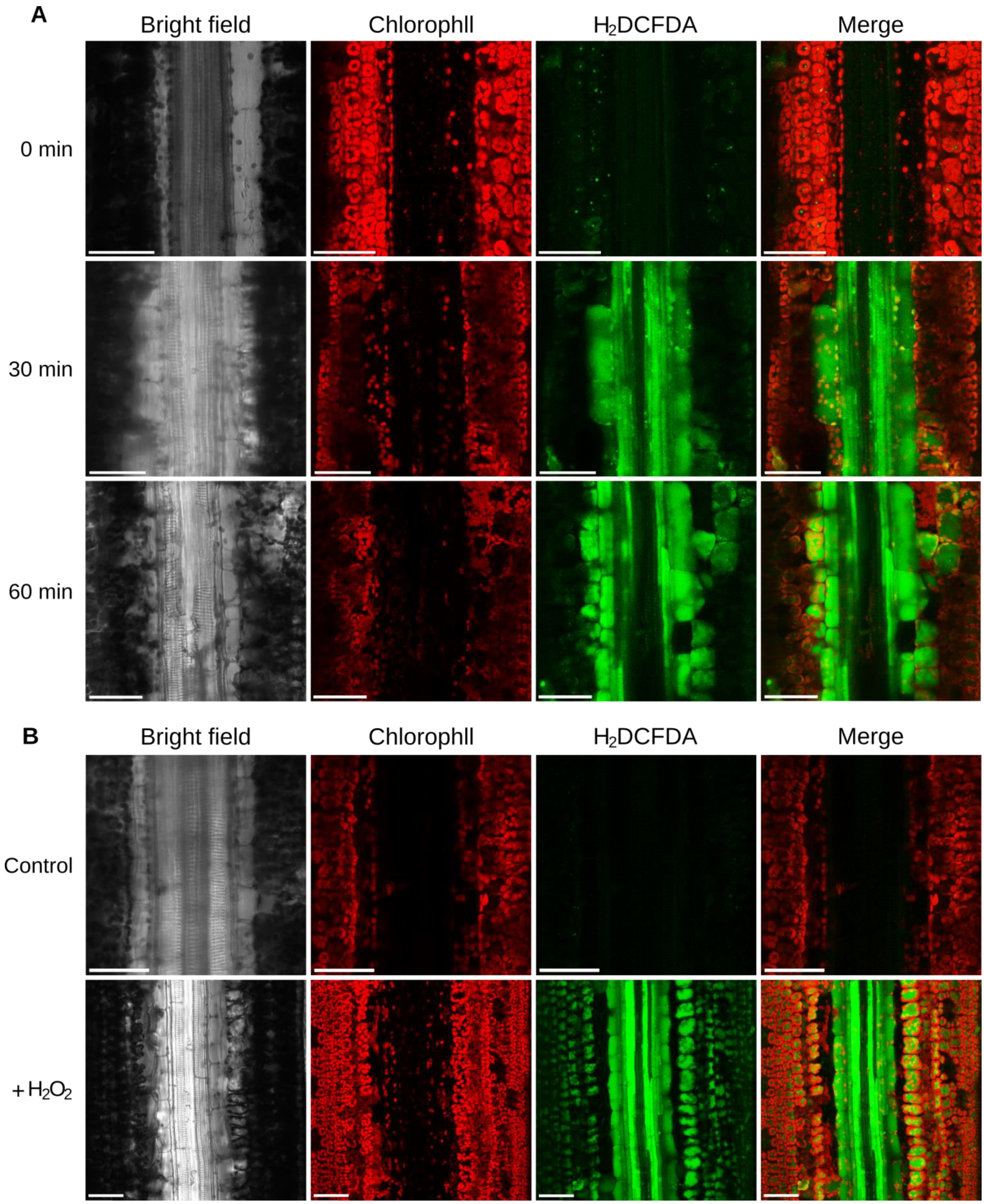

Figure S2. Confocal scanning microscopy indicates bundle sheath preferential fluorescence from H_2DCFDA in rice leaves exposed to high light. (A) Representative images of leaves were fed with 10 μM H_2DCFDA for 12 h prior to being exposed to high light (750 μmol m^{-2} s^{-1} photon flux density) for the times indicated. (B) Representative images from control leaves suppled with water as well as those suppled with 10 μM H_2DCFDA and exogenous 100 mM H_2O_2. Fluorescence from H_2DCFDA was not detected in control leaves, but in those to which H_2O_2 had been added it was detected in both mesophyll and bundle sheath strands. Scale bars denote 50 μm.

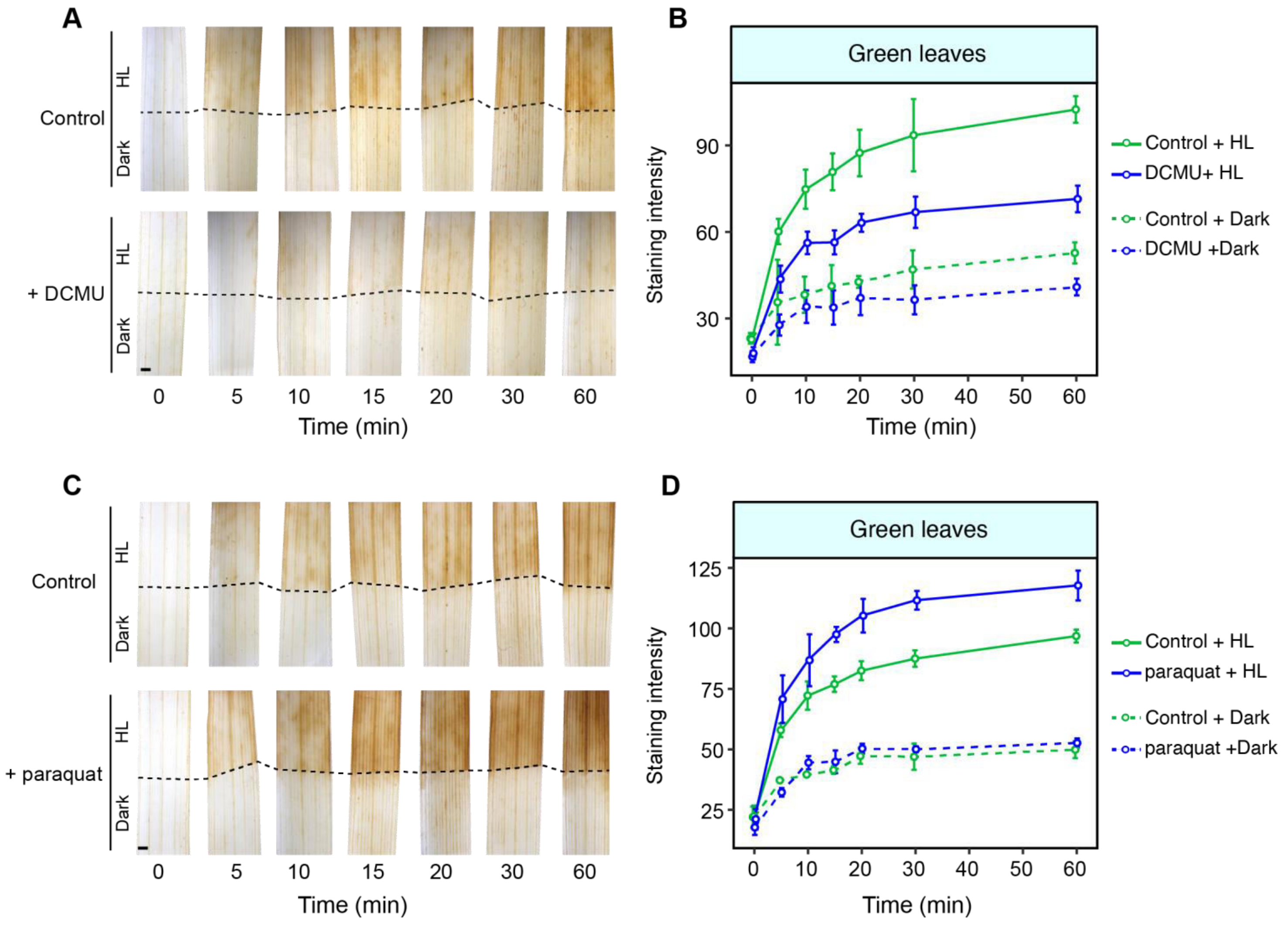

Figure S3. Photosynthesis contributes to ROS accumulation during high light treatment. (A&B) DCMU (100 μM) inhibits DAB accumulation in green leaves. (C&D) Paraquat (100 μM) increases accumulation of DAB in green leaves. ANOVA indicated a statistically significant reduction in DAB staining after DCMU treatment in green leaves ($p<0.001$) and increase after paraquat treatment in green leaves ($p<0.001$). Scale bars represent 1 mm.

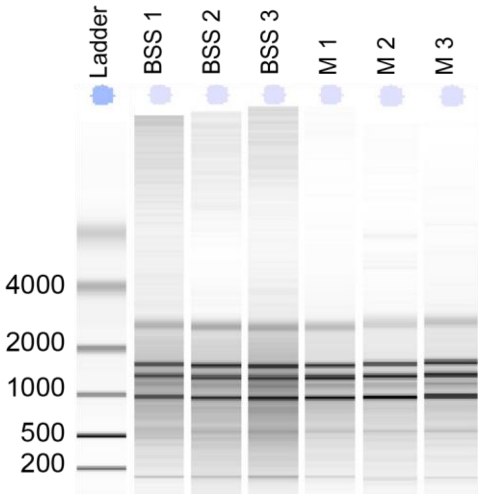

Figure S4. Electropherogram showing RNA quality.

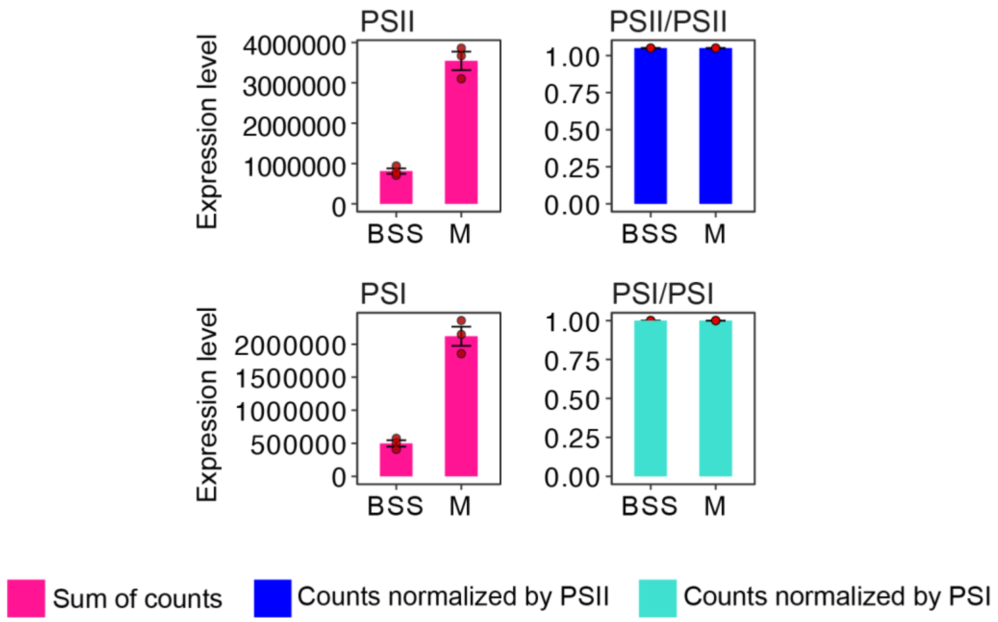

Figure S5. Normalisation of PSII and PSI indicates the expected one-to-one relationship in transcript abundance.

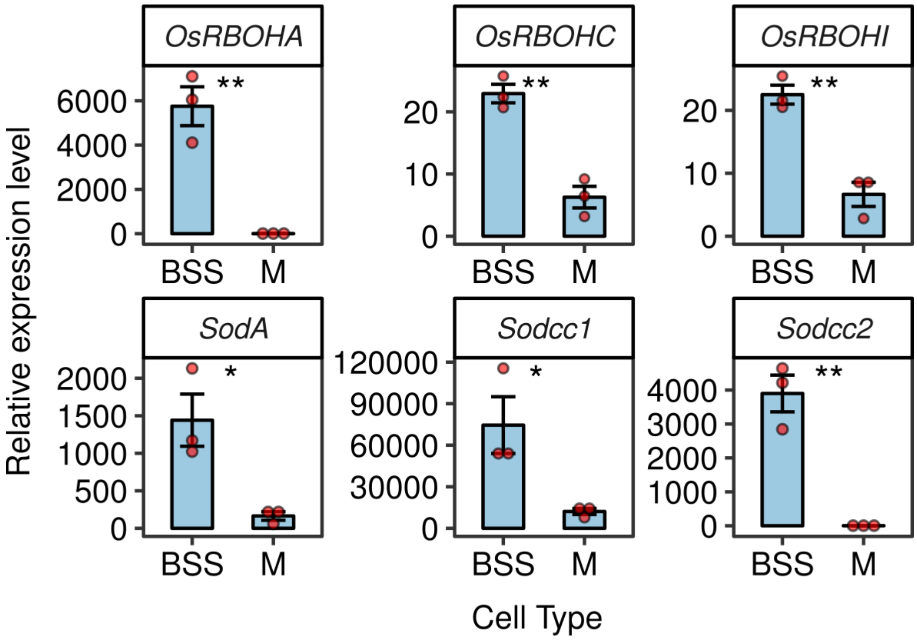

Figure S6. qPCR confirms analysis from deep sequencing and shows that transcripts of *RBOHA, RBOHC* & *RBOHI* as well as *SODA, SODCC1* & *SODCC2* are more abundant in bundle sheath stands (BSS) than mesophyll (M) cells. RNA extracted from green leaves. Data are normalized to *OsUBQ5* as a control and presented as mean ± one standard error, n = 3. T-tests indicate statistically significant differences (** $p<0.01$, * $p<0.05$).

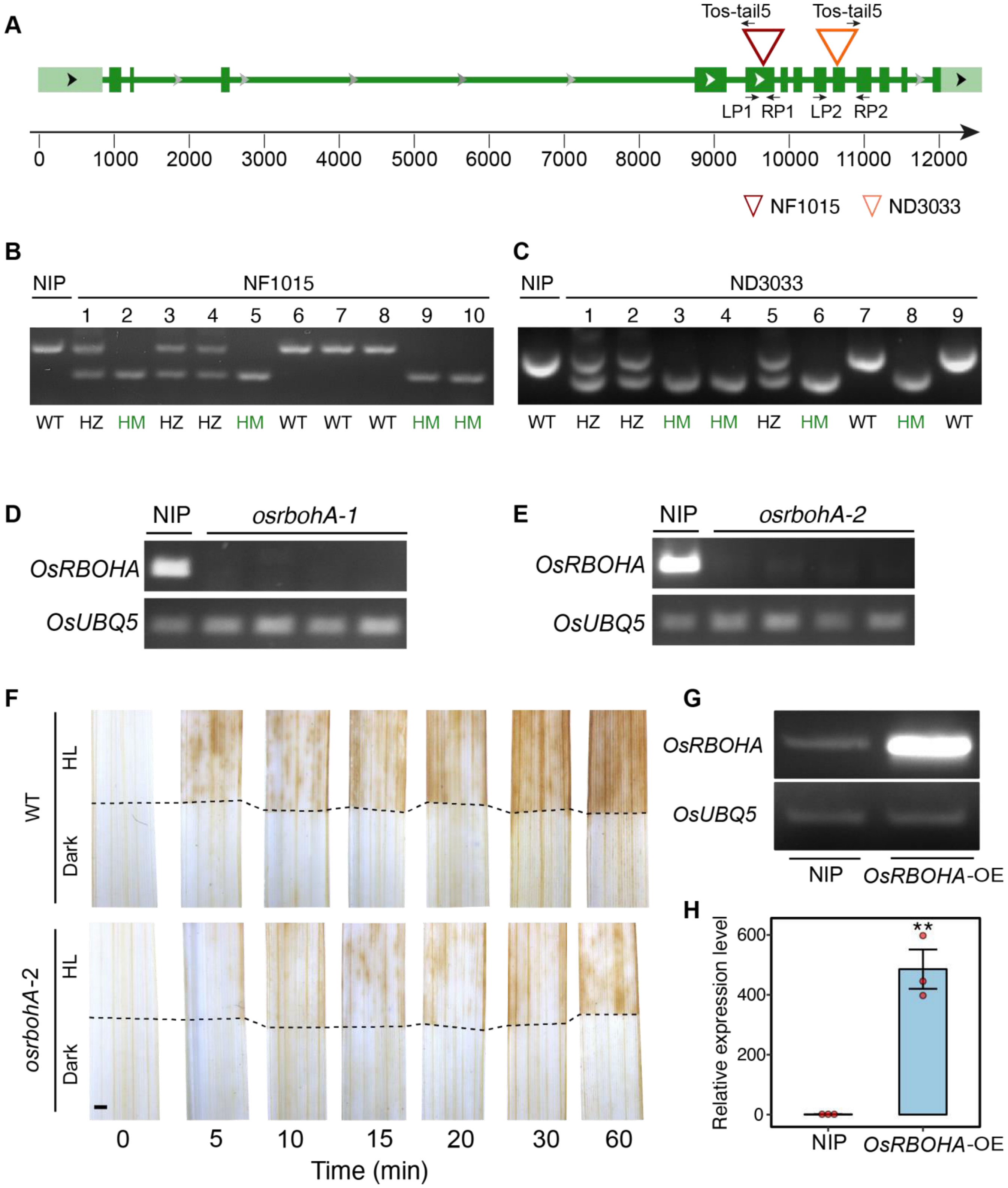

Figure S7. Genotyping of OsRBOHA mutants and over-expressors. (A) Diagram of the *OsRBOHA* allele. Red inverted triangle represents the Tos17 insertion position in line NF1015. Orange inverted triangle represent the Tos17 insertion position in line ND3033. Light green, dark green boxes and lines indicate UTRs, exons and introns respectively. (B) and (C) NF1015 and ND3033 were screened by PCR to identify homozygous mutants. Primers LP1, RP1 and Tos-tail5 were used for PCR of NF1015. Primers LP2, RP2 and Tos-tail5 were used for PCR of ND3033. HZ, heterozygosis. HM, homozygosis. (D) and (E) Semi-quantitative RT-PCR of NF1015 (*osrbohA-1*) and ND3033 (*osrbohA-2*) confirmed that *OsRBOHA* was not detectable. *OsUBQ5* was used as a control. NIP= Nipponbare. (F) Representative images showing that compared with wild type, green leaves of *osrbohA-2* have reduced DAB staining after high light treatment. Scale bar represents 1 mm. (G) and (H) Semi-quantitative RT-PCR and quantitative RT-PCR were used to confirm over-expression of *OsRBOHA* compared with wild type (WT). *OsUBQ5* was used as a control. T-test showed statistically significant difference in the over-expressor (OE) compared with wild type (WT) ($p<0.01$).

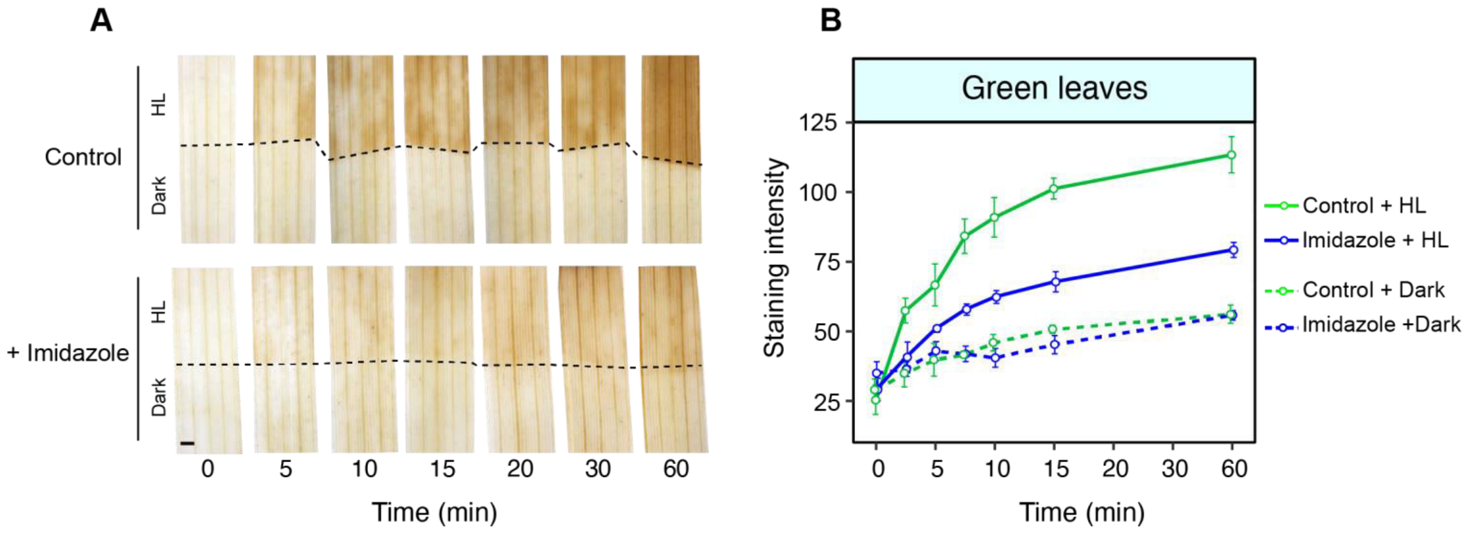

Figure S8. Inhibiting NAD(P)H oxidase activity with imidazole phenocopies the effect from diphenyleneiodonium in green leaves. (A) Representative images show Imidazole partially inhibits the accumulation of DAB in green leaves. (B) Semi-quantitation of DAB staining over time in green leaves infiltrated with or without imidazole. Scale bar represents 1 mm. (A). ANOVA showed DAB staining was reduced after Imidazole treatment in green leaves ($p<0.001$).

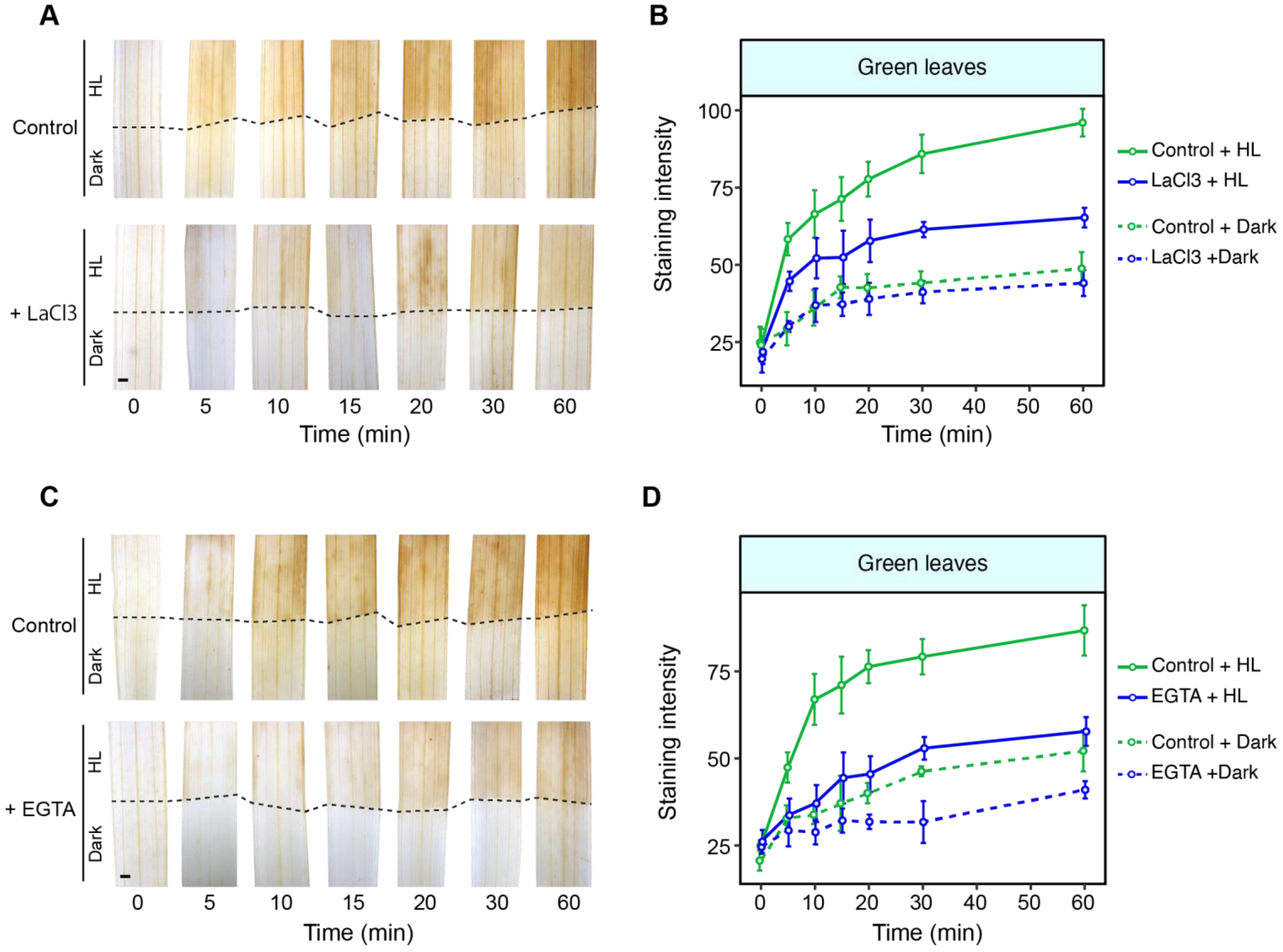

Figure S9. The calcium channel inhibitor $LaCl_3$ and Ca^{2+} chelator EGTA inhibit DAB staining in green leaves after high light treatment. (A&B) $LaCl_3$ partially inhibits the accumulation of DAB in green leaves. (C&D) EGTA partially inhibits DAB accumulation in green leaves. ANOVA confirmed a statistically significant reduction in DAB staining after $LaCl_3$ ($p<0.001$) and EGTA ($p<0.001$) treatment. Scale bars represent 1 mm.

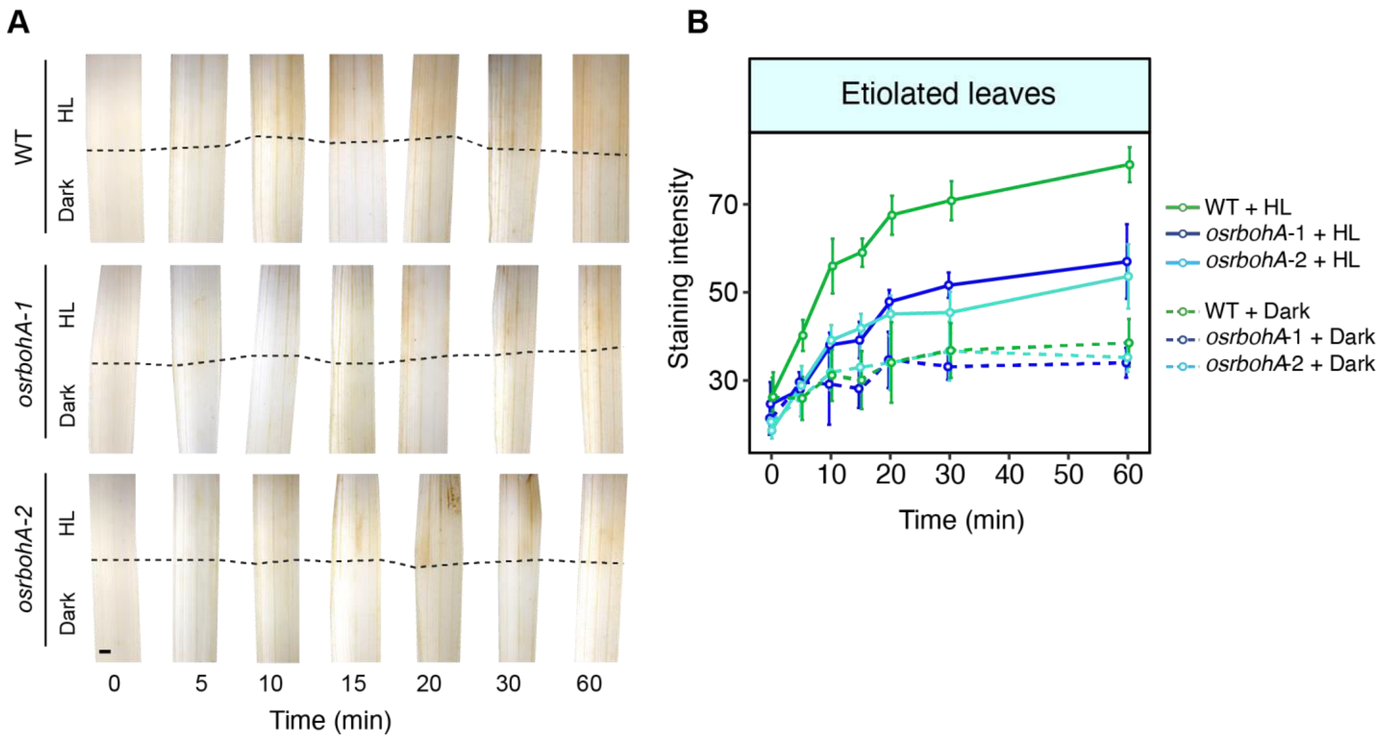

Figure S10. Knock-out of *OsRBOHA* reduced DAB staining in etiolated leaves under high light. (A) Representative images showing that compared with wild type, etiolated leaves of *osrbohA-1 & osrbohA-2* have reduced DAB staining after high light treatment. Scale bar represents 1 mm. (B) Semi-quantitation of DAB staining over time in etiolated leaves from wild type or *osrbohA-1* and *osrbohA-2* mutant alleles. ANOVA confirmed a statistically significant reduction in DAB staining of *osrbohA-1* ($p<0.001$) and *osrbohA-2* ($p<0.001$) compared to WT.

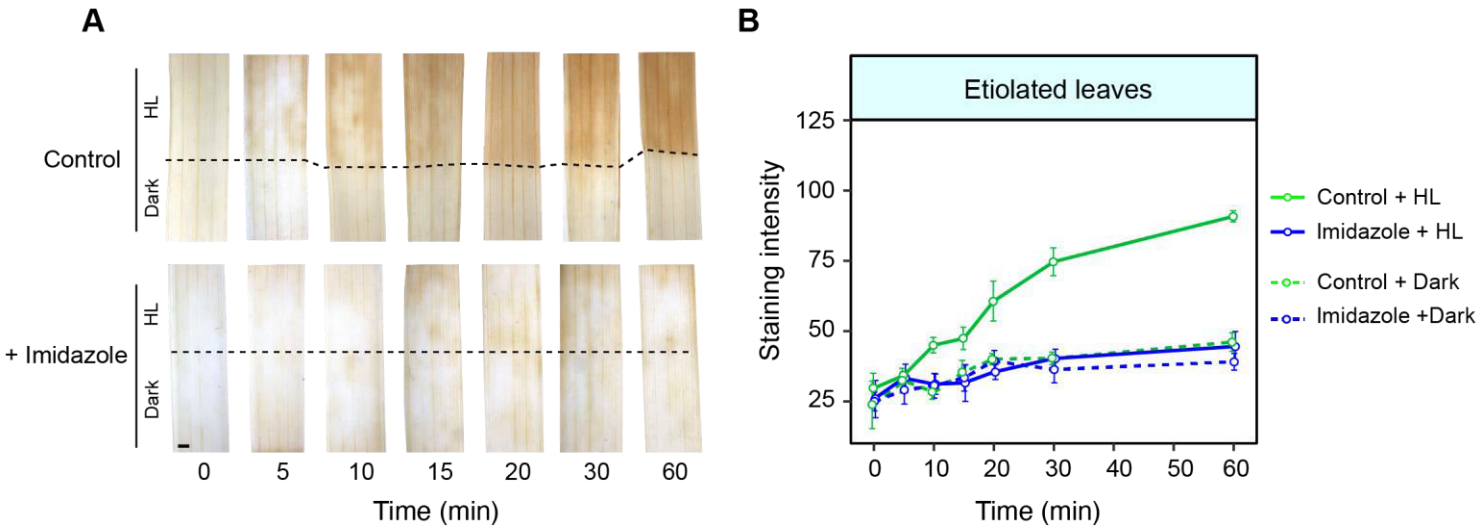

Figure S11. Inhibiting NAD(P)H oxidase activity with imidazole phenocopies the effect from diphenyleneiodonium in etiolated leaves. (A) Representative images show Imidazole partially inhibits the accumulation of DAB in green leaves. Scale bar represents 1 mm. (B) Semi-quantitation of DAB staining over time in etiolated leaves infiltrated with or without imidazole. ANOVA showed DAB staining was reduced after Imidazole treatment in etiolated leaves ($p<0.001$).

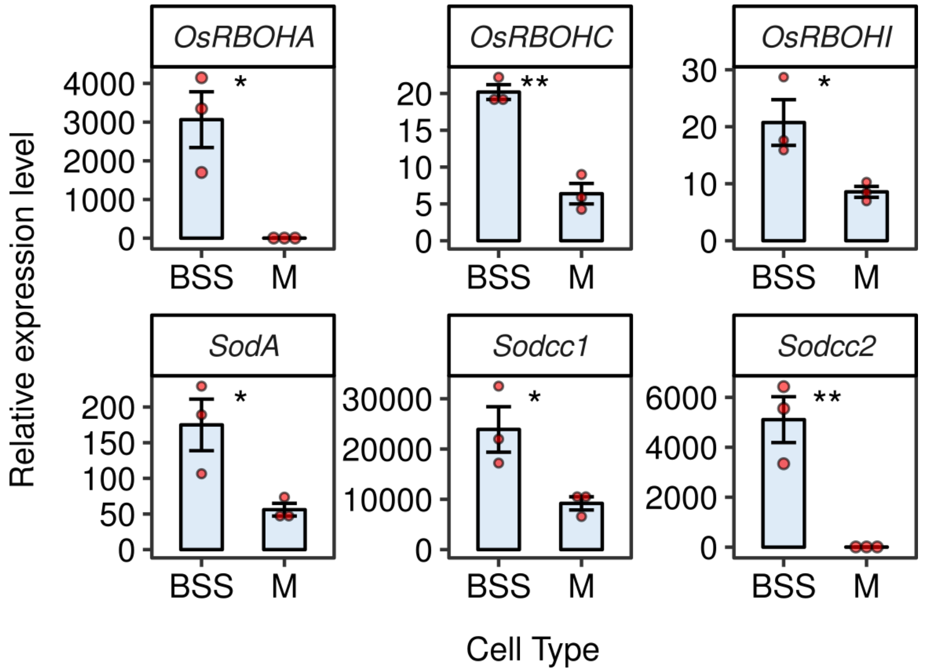

Figure S12. Q-PCR shows that transcripts encoding *OsRBOHA, OsRBOHC & OsRBOHI* as well as *SODA, SODCC1 & SODCC2* are more abundant in bundle sheath strands (BSS) than mesophyll (M) cells in etiolated leaves of rice. *OsUBQ5* was used to normalize expression of each gene, and data are presented as mean ± one standard error, n = 3. T-tests showed statistically significant differences between bundle sheath strands (BSS) and mesophyll (M) cells with ** = $p<0.01$, * = $p<0.05$.

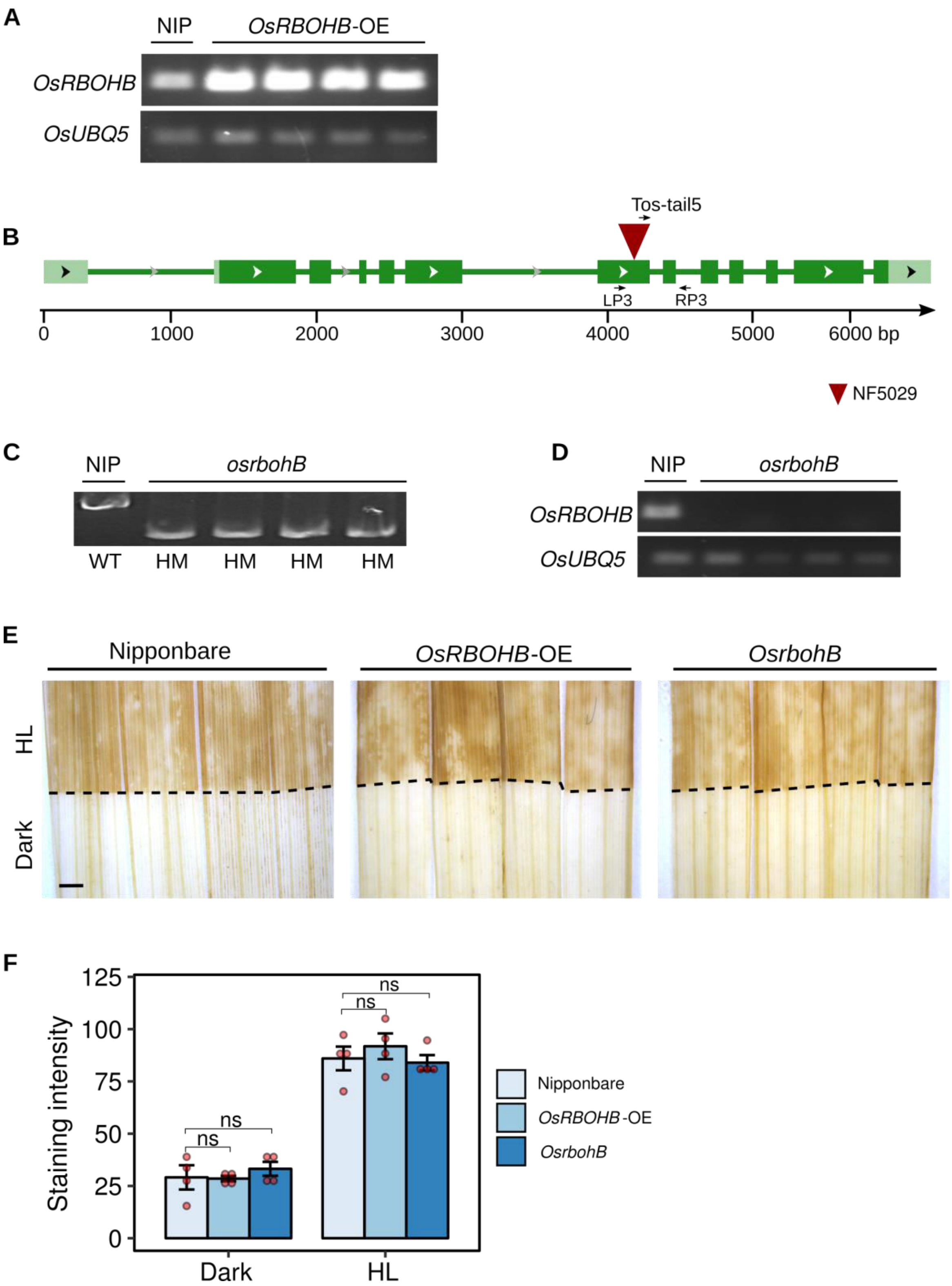

Figure S13. The *OsRBOHB* gene appears not to be involved in the high light response of the rice bundle sheath. (A) Semi-quantitative RT-PCR indicating that the over-expression line (*OsRBOHB-OE*) contains increased transcripts of *OsRBOHB* compared with wildtype (Nipponbare). *OsUBQ5* is shown below as a control. (B) Diagram of the *OsRBOHB*^{TOS17} allele with inverted triangle indicating the Tos17 insertion position in the line NF5029. Light green, dark green boxes and lines indicate UTRs, exons and introns respectively. (C) PCR confirmed the Tos17 insertion of NF5029 (*osrbohB)* plants. Primers LP3, RP3 and Tos-tail5 were used for PCR. (D) Semi-quantitative RT-PCR showed that *OsRBOHB* was not detectable in *osrbohB*. *OsUBQ5* was used as a control. (E) Representative images showing that the over-expression line and mutant allele of *OsRBOHB* show no difference in DAB staining compared with the Nipponbare control. (F) Semi-quantification of DAB staining in (E). Scale bar represents 2 mm. T-tests showed that there was no significant (ns) statistical differences associated with over-expression or knock-out of *OsRBOHB*.

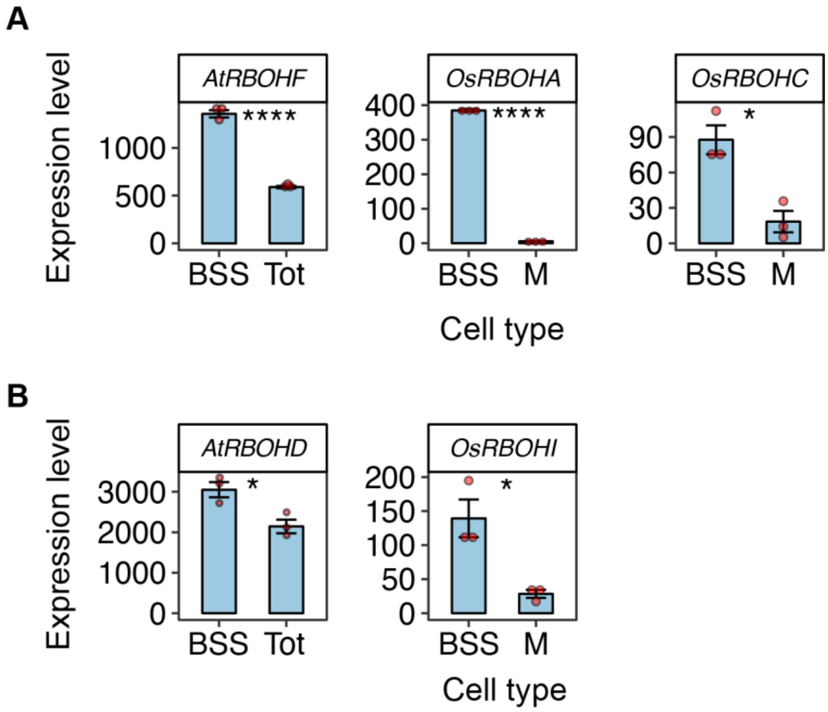

Figure S14. Orthologs of RBOHs in Arabidopsis show preferential expression in bundle sheath strands (BSS). (A) Transcript abundance of *AtRBOHF* in bundle sheath strands and total leaf (Tot) material of *A. thaliana,* and of *OsRBOHA* and *OsRBOHC* in bundle sheath strands and mesophyll (M) cells of rice. (B) Transcript abundance of *AtRBOHD* in bundle sheath strands and total leaf material or *A. thaliana* and of *OsRBOHI* in bundle sheath strands and mesophyll cells of rice. T-tests show statistically significant differences between cell types. **** = $p<0.0001$, * = $p<0.05$.
